# Effect of salt reduction interventions in lowering blood pressure: A comprehensive systematic review and meta-analysis of controlled clinical trials

**DOI:** 10.1371/journal.pone.0277929

**Published:** 2022-12-07

**Authors:** Soghra Aliasgharzadeh, Jafar Sadegh Tabrizi, Leila Nikniaz, Mehrangiz Ebrahimi-Mameghani, Neda Lotfi Yagin

**Affiliations:** 1 Student Research Committee, School of Nutrition and Food Sciences, Tabriz University of Medical Sciences, Tabriz, Iran; 2 Tabriz Health Services Management Research Center, Tabriz University of Medical Sciences, Tabriz, Iran; 3 Social Determinant of Health Research Center, School of Nutrition and Food Sciences, Tabriz University of Medical Sciences, Tabriz, Iran; Prince Sattam Bin Abdulaziz University, College of Applied Medical Sciences, SAUDI ARABIA

## Abstract

**Background:**

Excessive salt intake results in hypertension (HTN), which is a major risk factor for cardiovascular disease (CVD). This review and meta-analysis aimed to evaluate the effect of salt reduction interventions on systolic blood pressure (SBP) and diastolic blood pressure (DBP).

**Methods:**

Studies were identified via systematic searches of the databases, including PubMed, Embase, Scopus, and Web of Science. All the studies examining the effectiveness of salt reduction interventions on blood pressure (BP), regardless of age, sex, and HTN status, were included in the systematic review, and eligible studies were used in the meta-analysis. A random-effect model was applied for quantitative data synthesis.

**Results:**

A total of 50 trials extracted from 40 articles (21 trials on nutrition education,10 on self-help materials,17 on salt substitutes, and 2 on food reformulation) were included in the systematic review. The pooled results of 44 eligible trials showed that salt substitution and nutrition education interventions had significant effects on both SBP (WMD: -7.44 mmHg, P<0.001 and WMD: -2.75 mmHg, P<0.001, respectively), and DBP (WMD: -3.77 mmHg, P<0.001 and WMD: -2.11 mmHg, P<0.001, respectively). Furthermore, using self-help materials led to a significant reduction in SBP among subjects aged 25–60 years (WMD: -2.60 mmHg, P = 0.008); it also decreased both SBP and DBP among those who were hypertensive (WMD: -3.87 mmHg, P = 0.003 and WMD: -2.91 mmHg, P<0.001, respectively).

**Conclusion:**

Our results supported that salt substitution and nutrition education are effective nutrition strategies to lower BP. It seems that multi-component approaches could be more effective in improving BP status. However, further trials are required.

## Introduction

Cardiovascular disease (CVD) is the most important cause of morbidity and mortality throughout the world [1]. Among CVD risk factors, hypertension (HTN) is the most preventable one, with a global disability of 211 million every year [2, 3]. Furthermore, approximately 25% of the adult population is diagnosed with HTN worldwide, and it is expected that 29% (1.56 billion) of adults will be affected by 2025 [4]. New insights into HTN pathogenesis indicate that a range of risk factors contribute to the disease, and excessive salt intake is the most important one [5]. Moreover, observational studies have shown the critical role of salt intake in the development of HTN [6, 7]. Hence, the current World Health Organization (WHO) recommendation on salt intake for adults is a maximum of 5g/day; however, today’s food environment leads to too much salt intake, an average of 10g/day or further, causing an increase in BP [8]. In addition, robust evidence from randomized controlled trials (RCTs) shows that reducing dietary salt intake effectively reduces BP and consequently, reduces the number of premature deaths due to heart disease and stroke [9, 10]. Implementing different salt reduction initiatives in several countries, such as Finland, the UK, and China, resulted in a significant reduction in population salt intake [11]. Considering sufficient scientific evidence, population salt reduction has been identified by the WHO as a very cost-effective and feasible approach for HTN prevention and control and consequently CVD across the world [12, 13]. Although acceptable policy options, including individual-level and population-level interventions have been recommended to reach this goal, reducing salt intake efficiently is still a global problem [11]. In a recent meta-analysis by Jin et al. in China, four salt reduction strategies, including health education for behavior change, salt restriction diet, salt restriction spoon, and salt substitute on BP were evaluated. Their results showed that among studies with an RCT design, only salt substitute was an effective strategy in BP reduction by pooling effect of high-quality studies [14]. Furthermore, a systematic review by Hyseni et al. showed that in comparison to individual-based interventions, comprehensive policies including several components (food labelling, reformulation, and media campaigns) and population-wide strategies such as obligatory reformulation usually seem to reach greater reductions in salt consumption [15].

Although there is evidence to support the beneficial effects of some salt reduction intervention strategies on lowering BP, as far as the research investigated, no systematic review and meta-analysis studies have been conducted to critically evaluate and compare the effectiveness of various strategies in improving BP considering the role of baseline health condition, age category, geographical distribution, and the length of follow-up. Therefore, this study was carried out to examine the effect of different salt reduction strategies on BP reduction in all age groups and HTN status.

## Methods

This systematic review and meta-analysis was conducted in accordance with the recommendations by the Preferred Reporting Items for Systematic Reviews and Meta-Analyses (PRISMA) [16]. The completed PRISMA checklist is available in S1 Checklist. The study protocol was registered in PROSPERO (CRD42021274170).

### Search strategy and data sources

We systematically searched four electronic databases, including PubMed, Scopus, Embase, and Web of Science to find the studies evaluating the effect of community-based salt reduction strategies on BP reduction from 2000 to April 2022 with no country restriction. To construct the search strategy, a combination of relevant keywords identified from the exemplar paper as text words and the MeSH (Medical Subject Headings) terms from the PubMed database were used. The search terms included a combination of five sets of keywords: salt OR sodium AND intake OR consum* OR diet* AND reduc* OR curb OR limit OR restrict* OR minimi* OR eliminat* OR low OR free AND blood pressure OR hypertension AND polic* OR intervention OR strateg* OR initiative OR program OR action OR regulation OR activit* OR legislation OR ban OR law OR standard. For different electronic databases, different search strategies were adopted (see S1 Table).

All the identified articles were imported into the EndNote software (version X8, for Windows, Thomson Reuters, Philadelphia, PA, USA) for removing duplicates and assessing titles, abstracts, and full-texts for eligibility criteria. The bibliographies of the eligible articles and previous related review and meta-analyses were also manually checked to find additional relevant publications.

### Eligibility criteria and study selection

The articles were considered eligible if they fulfilled the PICO (population, intervention, comparator, and outcomes) criteria as follows: population (P): all age groups from all populations; intervention (I): the interventions with the aim of salt intake reduction including nutrition education programs with any combination of educational strategies such as dietary guidelines, nutrition counselling, salt reduction advise via phone message, e-mail or face-to-face counselling, cooking classes, group sessions, and distribution of educational material such as pamphlets about salt reduction, salt substitute with low sodium content, food labelling, food reformulation in terms of salt content, and self-help materials such as device delivery for monitoring daily sodium extraction and smartphone application; comparator (C): comparing the described interventions with standard care, placebo, or no intervention; outcome (O): measurement of SBP and DBP. Only RCTs, cluster RCTs, cross-over trials, and non-RCTs were included in this systematic review and meta-analysis.

We excluded all animal, before-after, and observational (including case-control, cohort, and cross-sectional) studies, as well as those with unavailable full texts. Moreover, studies in which salt reduction interventions had been implemented along with other interventions were not considered. Besides, publications without a control group or with insufficient data on the main outcome were excluded. There were no restrictions concerning the length of intervention or participants’ age and health status. For multiple publications that reported the same data, the article with the largest sample size was selected. To be included in the meta-analysis, the studies had to report the changes in BP mean and SD from baseline to the end of follow-up or any necessary data for computing them.

Titles and abstracts of all publications were initially screened by two independent reviewers (SA and LN). Next, ineligible articles were eliminated, and the reason for exclusion was noted. Then, the full-texts of relevant eligible articles were retrieved and re-evaluated. Finally, disagreements were resolved by discussion, or if necessary, further reviewers were consulted (MEM).

### Data extraction

The data extraction form was designed and adopted based on a template developed by the Editorial Resources Committee of the Cochrane Collaboration [17]. Two authors (SA and LN) independently extracted the following data from each eligible study: first author’s name, year of publication, country, study design, study population characteristics, the sample size of intervention and control groups, age, intervention type, intervention description, and details on the control group, follow-up duration, BP values before and after the intervention, and the main study results. For outcomes evaluating more than one time point, we extracted the latest follow-up data. Measures for intention-to-treat analysis were preferred to per-protocol when both were present. For two-arm crossover RCTs, only data from the first period were included due to the carry-out effect. For the studies with multiple groups, effect sizes for each intervention-control group were calculated separately. If data were not presented in a numerical format, they were extracted from figures using Web Plot Digitizer (https://automeris.io/WebPlotDigitizer/) [18]. Moreover, the study authors were contacted through email if the required data were not presented in the manuscript.

### Quality assessment

The quality of the included studies was evaluated by two independent researchers (SA and LN) using the Joanna Briggs Institute (JBI) Critical Appraisal Checklist for RCTs [19]. This instrument includes 13 criteria as follows: random sequence generation, allocation concealment, between-group similarity at baseline, blinding of participants, personnel, and outcome assessors, equal treatment in both groups other than the intervention of interest, identical outcome measurement in both groups, the detection of incomplete outcome data, intention to treat analysis, reliability of outcome measurement way, statistical analysis, and trial design appropriateness. Based on these items, each study was assigned to high, medium, or low quality. To be scored as high-quality, a study had to have at least two-thirds of applicable domains scored at ‘low risk’. In addition, random sequence generation, allocation concealment, and blinding of participants and personnel items were selected as the key domains to judge the overall risk of bias. Any disagreements between the evaluators were resolved by discussion or involvement of another author. All eligible studies were included in the current systematic review and meta-analysis regardless of the quality criteria.

### Statistical analysis

The pooled effects of salt reduction interventions on SBP and DBP were assessed using DerSimonian and Laird models to estimate the weighted mean differences (WMD), the difference in the change from baseline to follow-up in the intervention group versus the control group. When standard deviation (SD) for mean differences was not reported, it was calculated using the following formula: square root [(SD baseline)^2^ + (SD final)^2^ –(2R × SD baseline× SD final)]. Considering the wide methodological variability between the studies, random-effects models were applied to calculate the pooled effect size. The presence of between-study heterogeneity was evaluated using Cochrane’s Q test and I^2^ statistics. The statistical significance of the Q test and I^2^ >50% indicated the presence of heterogeneity [20]. To estimate the influence of individual studies on the overall pooled result, a sensitivity analysis was conducted using the leave-one-out method. To identify the possible sources of heterogeneity across studies, subgroup analyses were performed based on the quality of studies (high, moderate, low), participants’ age category (<25, 25–60, <60), participants’ HTN status (normotensives, hypertensives, a mixture of hypertensives and normotensives), follow-up duration (<2 months, 2–6.6 months, >6.6months), and sample size (<99, 99–242, >242). Moreover, meta-regression analysis was performed to explore the potential confounders (e.g., study quality, age category, hypertension status, follow-up duration, and sample size) that may have affected the main outcome of each intervention strategy. The presence of publication bias was examined using visual inspection of funnel plots, Begg’s adjusted rank correlation, and Egger’s regression asymmetry tests. All the analyses were conducted using Stata version 16.0 (StataCorp, College Station, TX, USA). A two-tailed P <0.05 was considered statistically significant.

## Results

### Study selection

**Fig 1:**
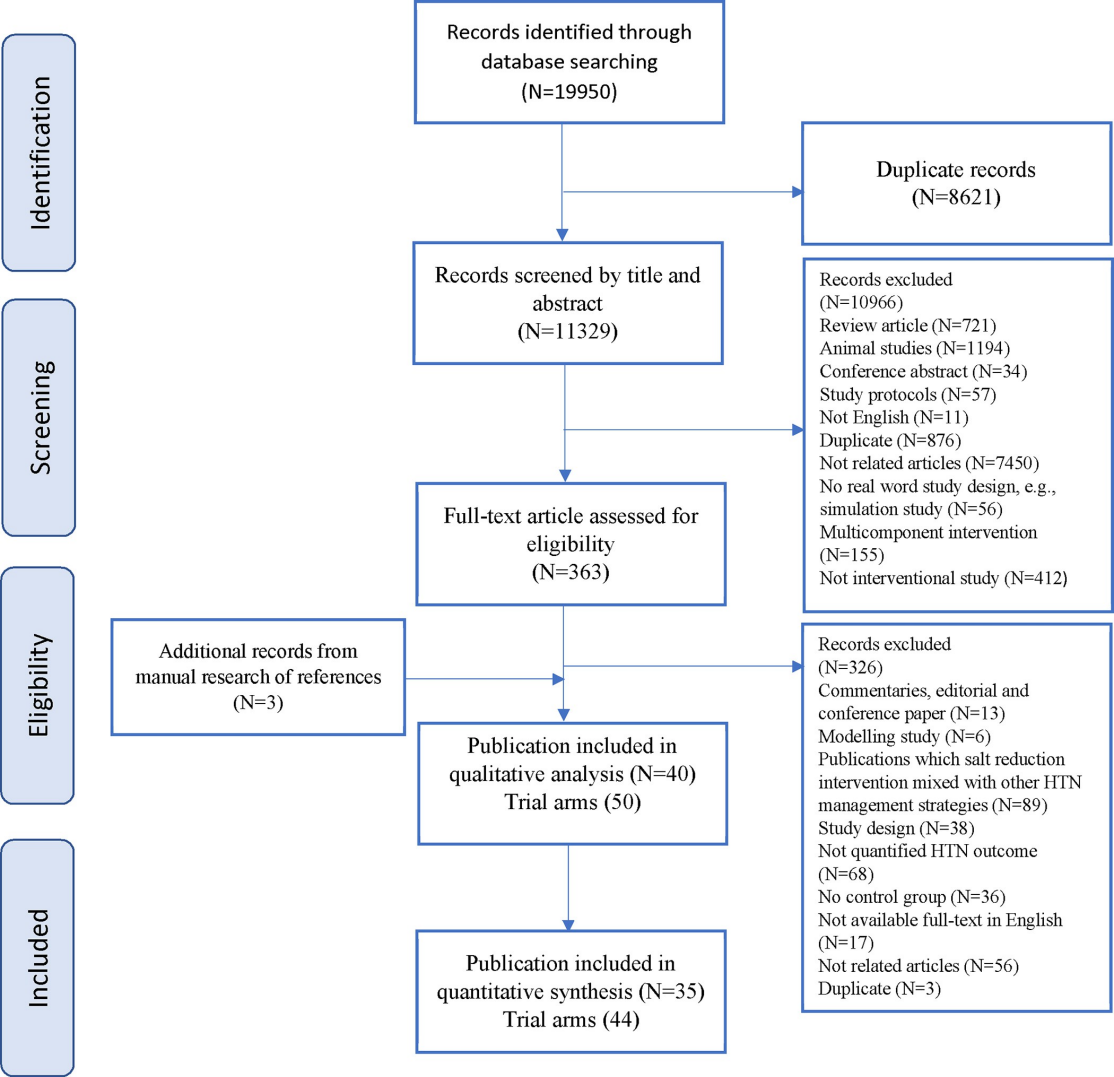
Flow diagram of the study screening and selection process.

The systematic search of the electronic databases resulted in a total of 19,950 potentially relevant articles. Fig 1 presents the PRISMA diagram of the study selection procedure. After eliminating 8,621 duplicates, the title and abstract of 1,329 remaining articles were screened, of which 1,966 irrelevant cases were excluded. Finally, 363 articles were assumed suitable for a detailed assessment of full‐text reports, of which 326 studies were excluded due to the following reasons: commentaries, editorial and conference papers (N = 13), modelling study (N = 6), salt reduction intervention mixed with other HTN management strategies (N = 89), study design not interested (N = 38), not quantitative HTN outcome (N = 68), no control group (N = 36), not available full-text in English (N = 17), not related articles (N = 56), and duplicate reports (n = 3). Three additional papers were identified through a hand search of references. In total, 40 studies with 50 trial arms were identified as eligible to be included in the systematic review. Of those, four trials were excluded because pre- or post-intervention BP data were not reported [21–24]. Besides, one study which had a stepped-wedge cluster randomized trial design was not included in the meta-analysis [25]. Finally, 44 trials in 35 articles fulfilled all the inclusion criteria for the meta-analysis.

### Study characteristics

Table 1 summarizes the characteristics of each included trial. Among the studies, three were conducted using a crossover design, while the remaining used a parallel design. Two studies randomized the participants into two intervention and one control group, and one RCT included four intervention and two control groups; all other studies used the conventional parallel two-group RCT design. The publication date of the articles was between 2005 and 2022. Of all the included studies, ten were carried out in the China [21, 23, 26–33], seven in Japan [34–40], three in India [41–43], two in France [44, 45], and one in Netherlands [46], United States [47], United Kingdom [48], Peru [25], Iran [49], Ireland [24], Italy [50], New Zealand [51], Indonesia [52], Bosnia and Herzegovina [53], Brazil [54], Australia [55], Portugal [56, 57], Finland [58], Argentina [22], Ghana [59], and Canada [60]. A total of 33,320 participants (17,218 in the intervention group and 16,102 in the control group), ranging from 34 [52] to 20,995 [33], were involved in the trials. The duration of the studies ranged from two weeks [22, 52] to five years [33]. Most of the studies included both genders, two recruited only females [34, 38], and one study included only males [39]. Of the 50 trials, 25 assessed the effects of the interventions among hypertensive participants [23, 24, 26, 29–31, 39, 42–50, 52–54, 58], three among normotensive individuals [30, 34, 35], and 22 among both hypertensive and normotensive participants [21, 22, 25, 27, 28, 31, 33, 36–38, 40, 41, 51, 55–57, 59, 60]. In 29 studies, BP was measured with an automatic sphygmomanometer, in five with a manual mercury sphygmomanometer [31, 40, 42, 49, 53], and six studies did not report BP measurement method [22, 30, 48, 51, 59, 60]. Furthermore, in all the studies, BP measurement was performed by health professionals or trained personnel, with the exception of three studies that relied solely upon self-reported measures [34, 35, 47]. The included studies employed different types of intervention strategies that were categorized into four domains; 21 trials with nutritional education [21, 28, 31, 38, 40–42, 46, 49, 50, 52, 55, 56, 59–61], 17 used salt substitutes [23, 25–27, 29–31, 33, 43–45, 54, 58], ten used self-help materials [34–37, 39, 47, 48, 51, 53, 57], and two trials employed food reformulation [22, 24]. The control groups of all trials received usual care or no intervention.

**Table 1:** Characteristics of the included studies.

First author (published year), country	Study design	Total number of participants/ sexes	Mean age (years)	Study population character	Assessment of blood pressure	Baseline SBP and DBP level (mm Hg)	Intervention type	Intervention description		Study duration	After intervention SBP and DBP level (mm Hg)	Main results outcomes	Quality
								Intervention group	Control group				
Silva-Santos (2021), Portugal	RCT	114/ F, M I:57 C:57	I: 47 ± 10 C: 49 ± 11	Adults older than 18 years	Portable sphygmomanometer: an average of the values of the two measurements	I: 126.2 ± 18.49, 79.7 ± 12.13 C: 127.7 ± 18.68, 81.2 ± 12.32	Self-help material	Using the Salt Control H equipment by the participants at home to control salt quantity for cooking all meals + provision Portuguese food guide	Provision Portuguese food guide	8 weeks	I: 123.0 ± 16.17, 74.9 ± 10.98 C: 124.1 ± 15.98, 76.4 ± 10.78	SBP and DBP decreased in both groups. The mean difference for SBP was significant in the control group and for DBP, the mean difference was significant in both groups	High
Neal (2021), China	C-RCT	20995/ F, M I:10504 C:10491	I: 65.2 ± 8.5 C: 65.5 ± 8.5	Adults who had a history of stroke or were 60 years of age or older and had poorly controlled blood pressure	Omron electronic sphygmomanometer: the average of the two measurements	I: 153.8 ± 23.4, 89.1 ± 14 C: 154.2 ± 23.6, 89.4 ± 14	Salt substitute	Free of charge replacement of regular salt with salt substitute contained 75% of NaCl, and 25% of KCl	Regular salt	5 years	I: 143 ± 20, 85 ± 12 C: 147 ± 21, 86 ± 12	• Significant difference in SBP, and DBP between two group after the intervention	Medium
Yu (2021), India	RCT	502/ F, M I: 252 C:250	I: 61.5 ± 11.1 C: 61.6 ± 12.0	Adults with diagnosed hypertension	Automated digital BP monitor: the mean of the last 2 sitting measurements	I: 132.8 ± 20.3, 83.7 ± 12.0 C: 132.1 ± 22.5, 82.9 ± 13.1	Salt substitute	Replacement of regular salt with salt substitute contained 70% of NaCl and 30% of KCl	Regular salt	3 months	I: 127.6 ± 19.6, 83.0 ± 12.6 C: 131.7 ± 21.7, 83.7 ± 13.3	• Significant reduction in SBP (an average reduction of 4.6 mmHg, P < 0.001) and DBP (1.1 mmHg, P = 0.02) in DBP in intervention group	High
He (2021), China	C-RCT	592/ F, M I: 297 C:295	I: 8.59 ± 0.34 C: 8.58 ± 0.47	School children	Validated automatic machine with the appropriate size of cuff: the average of the last two of the three readings	I: 92.8 ± 9.7, 64.3 ± 8.5 C: 92.5 ± 8.7, 64.0 ± 8.2	Education	Salt reduction education and monitoring via the app-based platform (AppSalt), complemented by the creation of supportive environments (eg, salt awareness posters put up in classrooms, campuses, and canteens) and seminars for both children and adults organised by teachers	-	12 months	I: 94.3 ± 9.9, 64.5 ± 7.8 C: 95.3 ± 8.7, 65.5 ± 7.4	• Blood pressure showed an increase from baseline to the end of the trial in both groups, and the mean effect comparing the intervention group with the control group was −1.19 mm Hg (−2.76 to 0.38) for SBP and −1.28 mm Hg (−2.64 to 0.09) for DBP	High
		1184/ F, M I: 594 C:590	I: 46.73 ± 13.06 C: 44.86 ± 12.61	Children’s adult family members	Validated automatic machine with the appropriate size of cuff: the average of the last two of the three readings	I: 132.8 ± 20.3, 83.7 ± 12.0 C: 118.9 ± 16.8, 77.5 ± 10.2	Education		-	12 months	I: 115.7 ± 16.5, 74.7 ± 9.9 C: 118.5 ± 17.5, 76.6 ± 11.0	• Blood pressure decreased in both groups, but the extent was greater in the intervention group and the mean effect was −2.53 mm Hg (−3.90 to −1.17) for SBP and −1.19 mm Hg (−2.15 to −0.23) for DBP	
Payne Riches (2021), United Kingdom	RCT	47/ F, M I: 31 C:16	I: 64 ± 12 C: 67 ± 7	Patients with an elevated blood pressure	-	I: 134 ± 16, 81 ± 10 C: 137 ± 15, 80 ± 8	Self-help material	Behaviour change advise session with provision of the SaltSwap app to help individuals identify lower-salt options when grocery shopping and provide feedback on swaps made + usual care	Usual care in the form of a postal copy of the publicly available British Heart Foundation Cut Down on Salt booklet or its successor Taking Control of Salt	6 weeks	I: 133.2 ± 16.9, 80.2 ± 10.7 C: 136.2 ± 15.9, 82.0 ± 10.5	• No significant effects on blood pressure • No significant between-group difference	Medium
Dorsch (2020), United States	RCT	50/ M, F I:24 C:26	I: 56.6 ± 10 C: 58.2 ± 11	Adults aged ≥18 years who were under treatment for hypertension	Automated blood pressure monitor: An average of 3 measurements was used as the participants’ blood pressure	I: 129.1 ± 20, 84.4 ± 12 C: 128.3 ± 14, 81 ± 8	Self-help material	Using LowSalt4Life, a just-in-time adaptive mobile app intervention that recommends lower dietary sodium food alternatives at home, restaurants, and grocery stores	Usual dietary advice	8 weeks	I: 121.52 ± 7.2, 80.73 ± 5.33 C: 127.78 ± 7.08, 81.56 ± 5.25	• The SBP change from baseline to week 8 in the App group was –7.5 mmHg while that in the No App group was –0.7 mmHg (P = .12)	Medium
Humalda (2020), Netherlands	RCT	99/ F, M I:52 C:47	I: 55.1 ± 11.5 C: 58.2 ± 13.2	Patients with chronic kidney disease (CKD)	Automated oscillometric device: the mean of the second and third reading	I: 139.6 ± 18.03, 83.9 ± 10.09 C: 139.2 ± 17.82, 83.3 ± 10.28	Education	Routine care plus a web-based self-management dietary sodium reduction intervention delivered through individual e-coaching and group meetings	Routine care	9 months	I: 131.5 ± 18.03, 79.2 ± 10.82 C: 135.3 ± 17.82, 80.1 ± 10.28	• Significant reduction in SBP and DBP at baseline to 9 months postbaseline in the intervention group • No significant difference in BP between groups after intervention	High
Bernabe-Ortiz (2020), Peru	Stepped-wedge cluster randomized trial	2376/ F, M I: 3605.3 person-years C: 2547.2 person-years	43.3 ± 17.2	Community-based Adults older than 18 years with no heart disease	Automatic blood pressure monitor: the average of the second and third measurements	-	Salt substitute	Replacement of regular salt with salt substitute contained 75% of NaCl and 25% of KCl	Common salt	6 months	-	An average reduction of 1.29 mm Hg (95% CI: −2.17, −0.41) in SBP and 0.76 mm Hg (95% CI: −1.39, −0.13) in DBP	High
Yasutake (2019), Japan	RCT	124/ F I:62 C:62	Total: 20.8 ± 0.9 I: 20.9 ± 0.8 C: 20.7 ± 0.9	Female college students without hypertension	Digital automatic sphygmomanometer: mean of 4 consecutive measurements	I: 100.9 ± 7.7, 63.2 ± 6.5 C: 101.9 ± 7.3, 63.9 ± 6.6	Self-help material	Provision of self-monitoring urinary salt excretion measurement device	The control group was asked to behave as they usually did	4 weeks	I: 100.9 ± 8.1, 62.2 ± 7.0 C: 99.3 ± 6.9, 61.5 ± 6.7	• No difference in SBP and DBP at end line in both groups • Significant change in SBP and DBP from baseline to end line in the control group	Medium
Rahimdel (2019), Iran	RCT	140/ M, F I:70 C:70	Total: 42.5 ± 5.2 I: 42.43 ± 4.9 C: 42.61 ± 5.5	Subjects at risk of developing hypertension (having SBP and DBP of 120–139 mmHg and 80–89 mmHg respectively).	Mercury sphygmomanometer: mean of 2 readings	I: 126.47 ± 6.9, 75.32 ± 8.1 C: 126.82 ± 7.1, 73.68 ± 7.2	Education	Education program based on the theory of planned behavior (TPB) for salt intake reduction	No intervention	3 months	I: 127.6 ± 6.5, 76.59 ± 7.4 C: 125.83 ± 10.8, 74.66 ± 9.7	• No significant change in SBP and DBP from baseline to end line in either group • No significant difference in change of SBP and DBP between two group	Medium
Cashman (2019), Ireland	Cross-over RCT	97/ M, F I:47 C:50	47.8 ± 9.3	Apparently healthy adults with slightly to moderately elevated BP (pre-hypertension to stage 1 hypertension: minimum >120/80 mmHg)	Automatic digital blood-pressure monitor: the average of the last two measurements	Not reported	Food reformulation	The replacement of bread and a limited number of other foods with equivalent foods which had lower salt content	Usual-salt diet	5 weeks	I: 131.0 ± 11.0, 84.6± 8.2 C: 134.3± 12.1, 84.7±8.5	• Significantly lower SBP during the reduced-salt dietary period compared to the usual-salt dietary period (by 3.3 mmHg on average; p < 0.0001) • No significant difference in DBP	Medium
Yasutake (2018), Japan	RCT	123/ M, F I:61 C:62	Total: 58.1 ± 17.4 I: 56.7 ± 17.5 C: 59.6 ± 17.5	Community-based Adults older than 21 years Without history of hypertension	Digital, upper-arm, automatic BP measuring instrument: mean of 4 consecutive measurements	I: 124.3 ± 15.6, 76.1 ± 10.8 C: 124.4 ± 18.3, 74.3 ± 10.9	Self-help material	Self-monitoring of 8h overnight urine extraction using the urinary salt excretion measurement device	No intervention	4 weeks	I: 123.9 ± 18.1, 75.7 ± 10.1 C: 126.4 ± 17.6, 73.9 ± 9.3	• No significant change in BP in either group • No significant difference between the two groups in term of SBP and DBP at end line	Medium
Yang (2018), China	RCT	51/ M, F I:24 C:27	I: 67.8 ± 5.34 C: 65.9 ± 6.17	Hypertensive patients with isolated systolic hypertension	The average of the two measurements	I: 161 ± 11.0 80.6 ± 4.94 C: 157 ± 10.4 81.3 ± 4.97	Salt substitute	Replacement of regular salt with salt substitute contained 65% NaCl, 30% KCl, and 5% calcium	Normal salt	6 months	I:153 ± 7.51 80.4 ± 5.92 C:159 ± 10.7 81.6 ± 5.00	• Significant reduction in SBP in intervention group after follow-up	Medium
		75/ M, F I:38 C:37	I: 67.3 ± 5.62 C: 65.4 ± 6.75	Hypertensive patients with non-isolated systolic hypertension	The average of the two measurements	I: 159 ± 12.2 85.0 ± 8.82 C: 157 ± 13.8 83.8 ± 8.21	Salt substitute	Replacement of regular salt with salt substitute contained 65% NaCl, 30% KCl, and 5% calcium	Normal salt	6 months	I: 155 ± 14.5 84.4 ± 9.00 C:159 ± 13.1 85.9 ± 9.00	• No changes in SBP and DBP in either the intervention or control groups	
Takada (2018), Japan	C-RCT	158/ M, F I:79 C:79	I: 62.0 ± 13.0 C: 63.9 ± 11.8	Family based adults aged 20 years old or older	Automatic monitor: mean of 2 readings	I: 138.2 ± 21.2, 77.8 ± 11.6 C: 138.5 ± 19.8, 77.3 ± 12.3	Self-help material	Holding lecture and handed out leaflets about salt reduction + Provision of self-monitoring device that estimates salt intake	Holding lecture and handed out leaflets about salt reduction	4 weeks	I: 135.0 ± 20.9, 77.6 ± 11.9 C: 138.2 ± 21.2, 77.8 ± 11.6	• Significant difference in SBP between two group after the intervention	High
Musso (2018), Italy	RCT	291/ M, F I:240 C:51	I: 63.2 ± 12.2 C: 64.1 ± 8.9	Patients on antihypertensive treatment	Digital Automatic BP Monitor	I: 144.5 ± 18.1, 85.9 ± 11.8 C: 145.4 ± 12.3, 84.5 ± 8.7	Education	Simple recommendations printed on a single A4 sheet of paper included on instruction to avoid salty foods and switch from regular bread to salt-free bread	Generic dietary advice	2 months	I: 135.7 ± 12.6, 80.8 ± 9.5 C: 143.3 ± 10.8, 85.3 ± 6.7	• Significant reduction in SBP and DBP in intervention group • Significant difference in SBP and DBP between two groups after intervention	Medium
Iwahori (2017), Japan	RCT	92/ M, F I:49 C:49	I: 55.0 ± 8.14 C: 54.0 ± 8.14	Healthy individuals or individuals with primary hypertension	Automated BP monitor: mean of 4 measurements	I: 125.9 ± 17.1, 79.3 ± 12.2 C: 125.8 ± 15.5, 77.9 ± 9.6	Self-help material	Provision brief dietary education and a leaflet as usual care + Provision of self-monitoring urinary salt excretion measurement device	Provision brief dietary education and a leaflet as usual care	1 months	I: 122.5 ± 17.6, 77.6 ± 12.0 C: 123.6 ± 14.8, 76.9 ± 9.6	• No significant difference in changes in SBP and DBP between two groups • No significant reduction in SBP and DBP after intervention	High
Borah (2018), India	RCT	393/ M, F I:199 C:194	I: 44.5 ± 14.4 C: 40.4 ± 15.5	Tea garden workers	Electronic BP monitor: average of last 2 reading	I: 140.4 ± 26.9, 84.8 ± 14.5 C: 141.6 ± 19.5, 84.2 ± 12.0	Education	Counselling (individual and group) and motivational campaign, meetings, posters, small booklet, health rally and audio-visual aids in the form of documentary film based on trans-theoretical model for behavior change	Appropriate medical advices as per existing medical practices for hypertensive participant	1 year	I: 134.6 ± 15.47, 77.6 ± 8.64 C: 141.1 ± 15.83, 83.3 ± 8.64	• Significant reduction in SBP and DBP in intervention group after follow-up • Significant different change in SBP and DBP between two groups	Medium
Eyles (2017), New Zealand	RCT	66/ M, F I:33 C:33	I: 64 ±7 C: 65±8	Adults with diagnosed cardiovascular disease and aged 40 years or over	Not reported	I: 131 ± 15, 80 ± 10 C: 134 ± 15, 79±9	Self-help material	Using SaltSwitch app: scanning the barcode of a packaged food using smartphone camera to receive an immediate interpretive, traffic light nutrition label on screen, along with a list of lower salt alternatives to ‘switch’ to.	access usual care cardiac rehabilitation services for people with CVD	4 weeks	I: 129 ± 11.5 C: 131 ± 11.5	• No significant difference in SBP between groups	High
Allaert (2017), France	RCT	41/ M, F I:22 C:19	Total: 51.0±16.0	Prehypertensive volunteers (120–139 mmHg SBP and/or 80–89 mmHg DBP) older than 18 years old and less than 70 years old	Fully automatic upper arm BP monitor: mean of 3 readings	I: 130.8 ± 5.7, 79.5 ± 6.8 C: 136.6 ± 10.3, 82.6 ± 7.8	Salt substitute	Provision of NaCl + Chitosan 3% (Symbiosal) plus a measuring spoon of a dose of 0.5 g of salt	Standard NaCl	8 weeks	I: 126.1 ± 6.5, 74.7 ± 6.4 C: 140.4 ± 8.3, 85.3 ± 9	• Significant decrease in SBP and DBP in intervention group • Significant difference in SBP and DBP between two groups at the end of intervention	High
Zhou (2016), China	RCT	462/ M, F I:224 C:238	I: 45.63 ± 13.72 C: 47.05 ± 13.46	Community-based patients with hypertension plus family members aged 18 years over	Automatic sphygmomanometer: mean of 2 readings	I: 154.02 ±28.26 91.46±14.75 C: 149.53 ± 23.67 89.07±13.78	Salt substitute	Replacement of regular salt with salt substitute contained 65% of NaCl, 25% of KCl and 10% MgSO2	Normal salt with 100% NaCL	3 years	I: 143.14 ±47.67 88.73 ±29.15 C: 148 ±55.64 90.99 ±29.78	• Significant different change in SBP and DBP between the two groups	High
Takada (2016), Japan	CRCT	35/ F I:18 C:17	I: 63.0 ± 10.3 C: 64.8 ± 11.5	Housewives aged 40 years over	Automatic BP monitor: average of 2 measurements	I: 135.6 ± 18.5 74.6 ±11.4 C: 131.9 ± 18.7 74.1±10.3	Education	Practical course for evaluating the amount of salt in a meal and instruction on salt-reduced cooking	lectures about healthy living	2 months	I: 138.0 ±17.6 76.8 ±9.08 C: 140.3 ±11.3 78.7 ±7.95	• No significant difference in SBP and DBP between two groups	Medium
Li (2016), China	C-RCT	2566/ F, M I: 1294 C:1272	I: 55 ± 15 C: 55 ± 14	Random sample of adults	Automated electronic sphygmomanometer: mean of 2 readings	Not reported	Education	Community-based health education through public lectures, and the display and distribution of promotional materials and availability of reduced-Na, added-K salt substitute at shops	usual practices without the introduction of any of the sodium reduction initiatives	18 months	I: 141 ± 22 86 ± 14 C:142 ± 23 86 ± 14	• No significant difference in SBP and DBP between two groups at the end of the study	Medium
Irwan (2016), Indonesia	RCT	34/ M, F I:17 C:17	67.9 ± 6.9 66.1 ± 5.7	Older people aged 60 years over with Hypertension or prehypertension	Sphygmomanometer	I: 147.5±17.3 88.0±12.8 C: 144.8±21.1 85.2±10.2	Education	Salt-reduction educational training, that applied self-care and self-efficacy theories (SRT)	Monthly health check-ups as usual care	2 weeks	I: 142.3±16.2 85.6±8.9 C: 143.5±16.9 87.3±7.5	• No significant difference in SBP and DBP among two groups after intervention	High
		34/ M, F I:17 C:17	65.8 ± 5.9 66.1 ± 5.7	Older people aged 60 years over with Hypertension or prehypertension	Sphygmomanometer	I: 145.5±30.5 87.5±15.5 C: 144.8±21.1 85.2±10.2	Education	Salt-reduction training and efficacy-maintenance meeting (SRTM)	Monthly health check-ups as usual care	1.5 months	I: 137.8±21.5 83.1±9.8 C: 138.5±19.3 85.7±10.6	• Significant reduction in SBP in SREM after training	
Pinjuh Markota (2015), Bosnia and Herzegovina	RCT	150/ M, F I: 76 C:74	I: 59.4 ± 13 C: 59.3 ± 12	Hypertensive adults	Mercury sphygmomanometry	I: 142.9 ± 20.6 84.7±10.3 C: 143.7±18.1 84.1±8.9	Self-help material	informational leaflets + Warning labels placed on home salt containers	individual information leaflet about the harmful effects of excessive salt intake	2 months	I: 137.6±16.1 81.8±8.5 C: 143.3±18.5 83.2±8.9	• Significant reduction in SBP and DBP in intervention group	High
He (2015), China	C-RCT	279/ M, F I:141 C:138	Total:10.1±0.5 I: 10.0±0.5 C: 10.2±0.5 Total:43.8±12	Schoolchildren	Automatic blood pressure monitor: mean of last 2 measurements	I: 106.2±11.79 67.0±12.97 C: 106.2±11.62 66.8±12.78	Education	Multiple theory-based education programme for behavior change to low-salt diet within the schools’ usual health education lessons	Usual health education lessons as in the curriculum	3.5 months	I: 110.0 ± 11.79 69.4 ± 12.97 C: 110.6 ± 11.62 70.2 ± 12.78	• No significant effect on BP in children	High
		553/ M, F I:278 C:275	.2 I:43.9±12.5 C:43.6±11.8	Children’s family members	Automatic blood pressure monitor: mean of last two measurementsل	I: 127.1±24.69 81.4±16.46 C: 124.1±24.23 79.9±16.15	Education	Transmitting messages through children and delivering educational materials in the form of a newsletter		3.5 months	I: 130.5±24.69 84.1±16.46 C: 129.1±24.23 83.2±17.77	• Significant mean effect of intervention on SBP in adults	
Barros (2015), Brazil SE	RCT	38/ F, M I:19 C:19	Total: 55.5 ± 7.4	Uncontrolled hypertensive patients	Semi-automatic Blood Pressure Monitor: (BP measured at least three times and at 1-minute intervals, until the differences between the measurements were lower than 4 mmHg) mean of last 2readings	I: 142.95±14.86 89.79±9.10 C: 143.44±13.99 91.19±9.10	salt substitute	Replacement of regular salt with the light salt contained (per gram) 130 mg of sodium, 346 mg of potassium and 44 mcg of iodine	Regular salt contained (per gram) 390 mg of sodium and 25 mcg of iodine	4 weeks	I: 127.11±15.64 75.95±9.47 C: 137.19±20.22 82.75±12.12	• Significant reduction in BP among intervention group after using light salt • Significant reduction in casual SBP among control group after using regular salt • Significant difference in casual BP between two groups at the end of study	Medium
Zhao (2014), China	RCT	282/ M, F I:141 C:141	Total:163.1 ± 11.2 I: 62.8 ± 11.1 C: 63.5 ± 11.3	Community-based hypertensives aged 40 years over	Automated electronic sphygmomanometer: mean of 3 readings	I: 176.1±22.4 103.2±12 C: 177.6±23.3 105.8±13.1	salt substitute	Free of charge replacement of regular salt with salt substitute contained 65% of NaCl, 25% of KCl and 10% MgSO2	Regular salt	3 months	I: 161.0 ±27.0 97.0 ±14.5 C: 170.2 ±26.8 102.6 ± 13.8	• Significant reduction in SBP and DBP in both groups • Significant net reduction of SBP and DBP in salt substitute treatment group in comparison with regular salt group	High
Petersen (2013), Australia	RCT	78/ F, M I:39 C:39	Total:63.1 ± 11.2 I: 62.9 ± 10.8 C: 61.6 ± 10.8	Hospital-based patients with T2DM aged 18 years over	Digital ambulatory blood pressure monitor: mean of 3 readings	I: 144 ±10.77 78 ±10.7 C: 138 ±16.15 76 ±10.77	Education	Provision of a single education session which focused on label reading	Usual diet	3 months	I: 136 ± 16.15 74 ± 10.77 C: 132 ± 16.15 73 ± 10.77	• No change in SBP and DBP during the intervention phase	Low
Cotter (2013), Portugal	RCT	F, M I:58 C:34	I: 10.9 ± 0.6 C: 10.8 ± 0.8	School children 10–12 years old	Automated oscillometric upper arm BP monitor: 3 separate measurements of BP were carried out at 2 min intervals. The average of the second and third measurements were considered	I: 115.1 ± 14.8 65.4 ± 8.2 C: 122.1 ± 14.1 73.5 ± 9.6	Education	Aromas school gardening club (2 h/week) plus regular lectures on the potential dangers of high salt intake	-	6 months	I: 111.3 ±11.6 64.8 ±7.4 C: 113.9 ±9.9 67.0 ±7.4	• No significant differences between the final values observed in the two groups and between baseline and final values in each group	Medium
		F, M I:47 C:34	I: 10.9 ± 0.7 C: 10.8 ± 0.8	School children 10–12 years old	Automated oscillometric upper arm BP monitor: 3 separate measurements of BP were carried out at 2 min intervals. The average of the second and third measurements were considered	I: 117.4 ±9.9 66.9 ± 8.0 C: 122.1 ± 14.1 73.5 ± 9.6	Education	Regular lectures on the potential dangers of high salt intake	-	6 months	I: 113.9 ±9.6 66.2 ±8.5 C: 113.9 ±9.9 67.0 ±7.4	No significant differences between the final values observed in the two groups and between baseline and final values in each group	
Allaert (2013), France	Cross-over RCT	40/ F, M I first:21 C first:19	I: 59.1 ±11.6 C: 58.0 ±12.7	Patient with stage 1 hypertension (SBP 140–159 mmHg or DBP 90–99 mmHg) aged 18 to 85 years	Homologated automated digital sphygmomanometers: the average of the 3 measurements	I: 149.2±4.9 93.4±3.0 C: 149.7±4.6 93.3±3.4	Salt substitute	lifestyle modifications and added salt reduction recommendation plus provision of NaCl + Chitosan 3% (Symbiosal)	lifestyle modifications and added salt reduction recommendation plus Standard NaCl	8 weeks	I: 136.1±9.5 82.2±7.7 C: 142.9±7.7 86.3±8.2	• significant greater reduction in SBP in the intervention group	High
		40/ F, M I:19 C:21	I: 58.0 ± 12.7 C: 59.1 ± 11.6	Patient with stage 1 hypertension (SBP 140–159 mmHg or DBP 90–99 mmHg) aged 18 to 85 years	Homologated automated digital sphygmomanometers: the average of the 3 measurements	I: 144.0±10.7 87.4±7.2 C: 137.1±9.4 82.3±6.9	Salt substitute	Provision of NaCl + Chitosan 3% (Symbiosal)	Standard NaCl	8 weeks	I: 140.7±11.5 84.8±9.2 C: 136.8±11.6 81.6±9.3	• No significant difference in SBP and DBP reduction between two groups	
Sarkkinen (2011), Finland	RCT	50/ M, F I:25 C:25	I: 57 ± 12 C: 54 ± 11	Subjects with mildly elevated BP (SBP in the range of 130–159 mmHg and/or DBP in the range of 85–99 mmHg) between 25–75 years old	Automatic sphygmomanometer: mean of two last readings	I: 140 ±13 89 ±8 C: 134 ±9 88 ±7	salt substitute	Replacement of salt used for cooking, salt in the main food sources of salt and table salt with smart Salt contained 50% NaCl, 25% KCl and 25% magnesal; [Mg4K(NH4)3Cl12·24H2O].	Regular salt consumption	8 months	I: 132 ±7 86 ±7 C: 138 ±9 90 ±7	• Significant reduction in SBP and DBP in the intervention group • Significant net reduction of SBP and DBP in smart salt group in comparison with regular salt group	Medium
Morikawa (2011), Japan	RCT	41/ M I:22 C:19	I: 48.3 ± 8.7 C: 47.1 ± 8.5	Hypertensive workers (SBP higher than 130 mmHg and/or DBP higher than 85 mmHg)	Automatic sphygmomanometer: mean of 2 readings	I: 149.8±14.3 96.9±11.3 C: 149.4±12.0 96.3±6.7	Self-help material	Group counselling on lifestyle modification from public health nurses and registered dietitians+ self-monitoring of 8h overnight urine extraction using the electronic salt sensor and sent personalized e-mail advice via cellular phone	Group counselling on lifestyle modification from public health nurses and registered dietitians	4 weeks	I: 144.4±12.2 90.7±10.2 C: 147.2±12.4 94.7±7.2	• Significant reduction in SBP and DBP after intervention compare to baseline in intervention group • Significant difference in change of DBP between two groups	Medium
Ferrante (2011), Argentina	Cross-over RCT	58/ M, F I:29 C:29	I: 41.5 ± 12.4 C: 37.1 ± 9.1	Normotensive or hypertensive adults aged 18 years or older	Average of 2 measurements	I: 114.4 ± 11.6 66.3 ±5.4 C: 113.8 ± 10.5 67.4 ± 6.1	Food reformulation	Consumption of low-salt bread contained 1.4% salt	Consumption of normal-salt bread contained 2.0% salt	15 days	-	• Significant reduction in SBP and DBP with the low-salt bread diet	High
Fujiwara (2010), Japan	Non-RCT	36/ M, F I:14 C:22	I: 69.0 ± 11.0 C: 75.1 ± 7.2	Outpatients with albuminuria no clinical features of RF, ischemic heart disease, or stroke	Mercury sphygmomanometer: mean of 2 measurements	I: 145.1±13.9 67.1±8.3 C: 134.9±13.1 66.4±12.2	Education	Holding a 30-minute session on dietary change for participant and their families at their home and 2-hour health promotion class for participants and their family and neighbors at a public town meeting hall	Usual care, which consisted of monthly visits and physician advice to reduce salt	3 months	I: 130.9 ±12.9 62.7 ±7.6 C: 130.9 ±14.0 66.8 ±7.8	• Significant reduction in SBP and DBP after intervention compare to baseline in intervention group • Significant difference in SBP reduction between two groups	Low
Zhou (2009), China	RCT	122/ F, M I:57 C:65	I: 68.1 ± 8.3 C: 65.4 ± 4.5	Community-based normotensive aged 50–80 years	Not reported	I: 125.0 ± 6.8 74.3 ± 6.4 C: 123.8 ± 7.0 74.5 ± 10.2	Salt substitute	Compound ion salt (CISalt) contained 65% NaCl, 30% KCl, 5% calcium, and 12mg/kg folic acid	Normal salt	6 months	I: 121.6 ± 7.4 71.7 ± 6.7 C: 127.4 ± 8.7 76.5 ± 5.9	• Continuous decrease of SBP and DBP over time with CISalt treatment • Significant between-group differences in SBP and DBP changes	High
		126/ F, M I:62 C:64	I: 67.5±5.2 C: 65.7 ±6.3	Community-based hypertensive aged 50–80 years	Not reported	I: 159.7 ± 11.7 83.3 ± 7.8 C: 157.7 ± 12.9 82.7 ± 7.1	Salt substitute	Compound ion salt (CISalt) contained 65% NaCl, 30% KCl, 5% calcium, and 12mg/kg folic acid	Normal salt	6 months	I: 150.4±10.7 78.8±8.6 C: 160.2±11.9 84±7.9	• Continuous decrease of SBP and DBP over time with CISalt treatment • Significant between-group differences in SBP and DBP changes	
Saptharishi (2009), India	RCT	58/ F, M I:28 C:30	I: 22.5 ± 1.47 C: 22.5 ± 1.4	Hypertensives and pre-hypertensives young adults	Mercury sphygmomanometer	I: 123.1 ± 8.1 83.7 ± 6.8 C: 123.8 ± 10.8 83.2 ± 7.2	Education	Practical suggestions on reducing salt intake to at least half of previous intake	No intervention	8 weeks	I: 120.8 ± 6.7 80.3 ± 5.3 C: 123.7 ± 10.4 82.8 ± 6.3	• Significant reduction in SBP and DBP in intervention group • No significant difference in SBP and DBP reduction between the two groups	Medium
Mu (2009), China	RCT	215/ F, M I:101 C:114	I: 20.3 ± 3.1 C: 21.4 ± 3.9	Adolescents with high BP (SBP ≥90th percentile by age and sex)	Mercury sphygmomanometer: mean of 3 readings	I: 123.8 ± 12.9 75.0 ± 11.1 C: 124.3 ± 14.1 77.0 ± 11.8	Salt substitute	Provision of salt which KCL and CaCl had been added	No interventions	2 years	-	• Significant reduction in SBP and DBP in the intervention group • Significant different change in SBP and DBP between two group	Medium
		588/ M, F I:334 C:254	-	Family members of hypertensive adolescent	Mercury sphygmomanometer: mean of 3 readings	-	Salt substitute	Provision of salt which KCL and CaCl had been added	No intervention	2 years	-	• Significant different change in SBP and DBP between two group	
		224/ M, F I:110 C:114	I: 20.6 ± 3.1 C: 21.4 ± 3.9	adolescents with high BP (SBP ≥90th percentile by age and sex)	Mercury sphygmomanometer: mean of 3 readings	I: 121.5 ± 12.8 75.4 ± 9.9 C: 124.3 ± 14.1 77.0 ± 11.8	Education	Health-behavior education about salt-restricted diet	No intervention	2 years	-	• Significant different change in SBP and DBP between two group	
		592/ M, F I:338 C:254	-	Family members of hypertensive adolescent	Mercury sphygmomanometer: mean of 3 readings		education	Health-behavior education about salt-restricted diet	No intervention	2 years	-	• Significant different change in SBP and DBP between two group	
CSSSC (2007), China	RCT	608/ M, F I:306 C:302	I: 59 ±10.0 C: 61 ± 9.7	Individuals with high risk of future vascular disease	Automatic sphygmomanometer: mean of 2 readings	I: 159 ± 25 93 ± 14 C:159 ± 26 93 ± 14	Salt substitute	Replacement of regular salt with salt substitute contained 65% of NaCl, 25% of KCl and 10% MgSO2	Regular salt	12-months	I: 141.61 ± 61 93 ± 14 C:148.04 ± 26 93 ± 14	• Significant mean difference in SBP between randomized groups • No significant differences between randomized groups for DBP	High
Cappuccio (2006), Ghana	CRCT	1013/ M, F I:522 C:491	I: 54 ± 11 C: 55 ± 11	Community-based individuals	Not reported	I: 129.2 ± 24.6 76.9 ± 13.0 C:125.6 ± 25.5 75.2 ± 13.3	Education	general health education + more specific education as to how to reduce salt intake	general health education	6 months	I: 127.9 ± 27.7 76.0 ± 14.2 C:127.4 ± 26.0 78.7 ± 14.3	• Significant different change in SBP and DBP between two group	Medium
Arcand (2005), Canada	RCT	50/ M, F I:25 C:25	I: 56 ± 3 C: 61 ± 3	patients with stable heart failure	Not reported	I: 114 ± 4 71 ± 2 C:114 ± 4 71 ± 2	Education	Prescription a 2 g/d sodium-restricted diet as a usual care provision of standardized nutrition education materials as a usual care + holding individualized nutrition-counselling appointments with a registered dietitian	Prescription a 2 g/d sodium-restricted diet and provision of standardized nutrition education materials as a usual care	3 months	I: 110 ± 4 68 ± 2 C:11 6± 4 74 ± 3	• No significant reduction in BP in both group	Medium

C: Control; I: Intervention; RCT: Randomized controlled-trial; CRCT: Cluster randomized controlled trial; F: Female; M: Male; DBP: Diastolic blood pressure; SBP: Systolic blood pressure

### Quality assessment

According to the quality criteria mentioned in the methods section, 18 studies were regarded as high-quality [22, 23, 25, 27–30, 36, 37, 43–46, 51, 53, 57, 61], 18 as moderate [21, 24, 26, 31, 33–35, 38, 39, 41, 42, 47–50, 54, 56, 58–60], and two studies as low-quality [40, 55]. All RCTs described the randomization, of which 4 studies had an inappropriate random allocation method [39, 40, 50, 54], and 17 studies did not provide precise information of randomization [24, 26, 34, 35, 38, 41, 44, 45, 55, 56, 58–60]. While 15 studies used allocation concealment to prevent selection bias [23, 27–30, 37, 43, 45, 46, 48, 51–53, 57, 61], eight studies did not mention it [38–41, 49, 50, 54, 55]; the other remaining studies did not clearly explain any procedure used to conceal the allocation. Most studies did not report adequate information to judge the participants, personnel, and outcome assessors’ blinding. Furthermore, in several trials, participants and personnel’s blinding was not completely applicable due to the nature of the intervention strategies, such as education and self-help materials. In addition, 29 articles that described dropouts and their causes and also group differences in terms of dropout were considered as low risk of bias [21–26, 28–30, 36–40, 42–45, 47, 48, 50–54, 56, 57, 60, 61]. Moreover, 11 studies had incomplete outcome data [27, 31, 33–35, 41, 46, 49, 55, 58, 59]. Twenty two studies employed the intention-to-treat approach to avoid the bias in their original analysis [21, 23–25, 28–30, 33, 36, 38, 41–46, 48, 51, 53, 57, 59, 61], five studies analyzed outcome data according to the per-protocol approach [34, 37, 52, 55, 58]; the remaining studies did not explain the participants’ number included in the final analyses. The detailed results of the quality assessment of the included studies are shown in Table 2.

**Table 2:** Quality assessment of included studies.

Quality assessment criteria	1. Was true randomization used for assignment of participants to treatment groups?	2. Was allocation to treatment groups concealed?	3. Were treatment groups similar at the baseline?	4. Were participants blind to treatment assignment?	5. Were those delivering treatment blind to treatment assignment?	6. Were outcomes assessors blind to treatment assignment?	7. Were treatment groups treated identically other than the intervention of interest?	8. Was follow up complete and if not, were differences between groups in terms of their follow up adequately described and analysed?	9. Were participants analysed in the groups to which they were randomized?	10. Were outcomes measured in the same way for treatment groups?	11. Were outcomes measured in a reliable way?	12. Was appropriate statistical analysis used?	13. Was the trial design appropriate, and any deviations from the standard RCT design (individual randomization, parallel groups) accounted for in the conduct and analysis of the trial?	*Quality score*	*Quality*
Silva-Santos, 2021	Y	Y	Y	NA	NA	U	Y	Y	Y	Y	Y	Y	Y	10	H
Neal, 2021	Y	U	Y	N	N	N	Y	U	Y	Y	Y	Y	Y	8	M
Yu, 2021	Y	Y	Y	U	Y	Y	Y	Y	Y	Y	Y	Y	Y	12	H
He, 2021	Y	Y	Y	NA	NA	U	Y	Y	Y	Y	Y	Y	Y	10	H
Payne Riches, 2021	Y	Y	Y	NA	NA	N	N	Y	Y	U	U	Y	Y	7	M
Dorsch, 2020	Y	U	Y	NA	NA	U	Y	Y	U	Y	N	Y	Y	7	M
Humalda, 2020	Y	Y	Y	NA	NA	U	Y	N	Y	Y	Y	Y	Y	9	H
Bernabe-Ortiz, 2020	Y	U	N	NA	NA	Y	Y	Y	Y	Y	Y	Y	Y	9	H
Yasutake, 2019	U	U	Y	NA	NA	U	Y	N	N	Y	Y	Y	Y	6	M
Rahimdel, 2019	Y	N	Y	NA	NA	U	Y	N	U	Y	Y	N	Y	6	M
Cashman, 2019	U	U	Y	N	N	U	N	Y	Y	Y	Y	U	N	5	M
Yasutake, 2018	U	U	Y	NA	NA	U	Y	N	U	Y	U	Y	Y	5	M
Yang, 2018	U	U	Y	U	U	U	Y	Y	U	Y	Y	Y	Y	7	M
Takada, 2018	Y	U	N	NA	NA	Y	Y	Y	Y	Y	N	Y	Y	8	H
Musso, 2018	N	N	Y	NA	NA	U	Y	Y	U	Y	Y	Y	Y	7	M
Iwahori, 2018	Y	Y	Y	NA	NA	U	Y	Y	N	Y	Y	N	Y	8	H
Borah, 2018	U	N	N	NA	NA	U	Y	N	Y	Y	Y	Y	N	5	M
Eyles, 2017	Y	Y	Y	NA	NA	N	Y	Y	Y	Y	Y	U	Y	9	H
Allaert, 2017	U	Y	U	Y	Y	U	Y	Y	Y	Y	Y	Y	Y	10	H
Zhou, 2016	Y	Y	N	Y	Y	U	Y	N	U	Y	Y	Y	Y	9	H
Takada, 2016	U	N	N	NA	NA	Y	N	Y	Y	Y	Y	Y	Y	7	M
Li, 2016	Y	U	U	NA	NA	N	Y	Y	Y	Y	Y	N	N	6	M
Irwan, 2016	Y	Y	Y	Y	Y	U	Y	Y	N	Y	Y	Y	Y	11	H
Pinjuh Markota, 2015	Y	Y	Y	NA	NA	U	U	Y	Y	Y	U	Y	Y	8	H
He, 2015	Y	Y	Y	NA	NA	Y	Y	Y	Y	Y	Y	Y	Y	12	H
Barros, 2015	N	N	Y	Y	N	N	Y	Y	U	Y	Y	Y	Y	8	M
Zhao, 2014	Y	Y	Y	Y	N	U	Y	Y	Y	Y	Y	Y	Y	11	H
Petersen, 2013	U	N	Y	NA	NA	N	Y	N	N	Y	Y	N	N	4	L
Cotter, 2013	U	U	Y	NA	NA	U	Y	Y	U	Y	Y	Y	Y	7	M
Allaert, 2013	U	U	Y	Y	Y	U	Y	Y	Y	Y	Y	Y	Y	10	H
Sarkkinen, 2011	U	U	Y	Y	Y	U	Y	N	N	Y	Y	Y	Y	8	M
Morikawa, 2011	N	N	Y	NA	NA	U	Y	Y	U	Y	Y	Y	Y	7	M
Ferrante, 2011	Y	U	Y	Y	Y	Y	Y	Y	U	Y	Y	Y	Y	11	H
Fujiwara, 2010	N	N	N	NA	NA	N	N	Y	U	Y	Y	Y	N	4	L
Zhou, 2009	Y	Y	Y	Y	N	U	Y	Y	Y	Y	U	Y	Y	10	H
Saptharishi, 2009	Y	U	N	NA	NA	U	Y	Y	Y	Y	U	Y	Y	7	M
Mu, 2009	Y	U	U	Y	U	U	Y	N	U	Y	Y	Y	Y	7	M
CSSSC, 2007	Y	Y	Y	Y	Y	Y	Y	Y	Y	Y	Y	Y	Y	13	H
Cappuccio, 2006	U	U	U	Y	NA	Y	Y	N	Y	Y	U	N	Y	6	M
Arcand, 2005	U	U	Y	NA	NA	U	Y	Y	U	Y	U	N	Y	5	M

### Effect of salt reduction interventions on SBP and DBP

**Fig 2:**
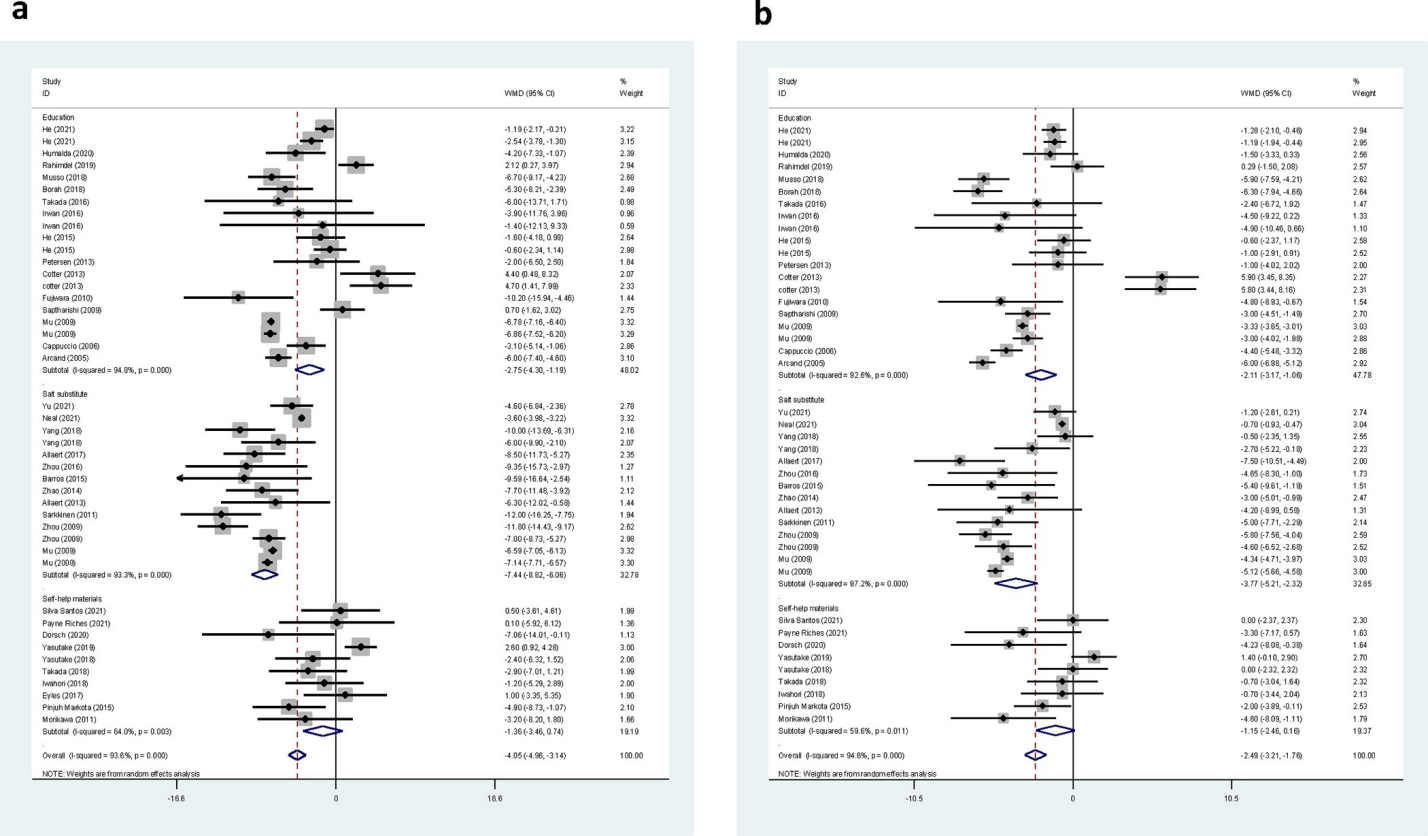
Forest plots of the overall effect of salt reduction intervention on SBP (a) and DBP (b).

Overall, the impact of the salt reduction interventions on SBP and DBP was evaluated in 50 and 49 trial arms, respectively. A brief explanation of each trial included in the current systematic review is presented in the next sections. The forest plots of the net changes in SBP and DBP from each trial were included in the meta-analysis, and the pooled net changes are presented in Fig 2(A) and 2(B). In 36 trials, the net change in SBP was in favor of the intervention groups; however, this change was significant only in 26 trials. The meta-analysis results revealed that salt reduction interventions significantly decreased SBP (WMD: -4.05 mmHg, 95% CI: -4.96, -3.14, P < 0.001) (Fig 2A). However, there was a significant between-study heterogeneity (I^2^ = 93.6%, P < 0.001). In terms of DBP, 37 studies reported a greater reduction in DBP in the intervention group compared to the control group, but this reduction was significant only in 24 trials. As shown in Fig 2B, the salt reduction interventions resulted in a significant decrease in DBP (WMD: -2.49 mmHg, 95% CI: -3.21, -1.76, P < 0.001). However, there was evidence of high heterogeneity (I^2^ = 94.6%, P < 0.001). Moreover, the sensitivity analyses using leave-one-out showed that the pooled effects of the interventions on outcomes were robust, which did not alter significantly by removing any single study.

### Effect of health education interventions on SBP and DBP

Twenty-one trials examined changes in BP following a health education intervention as a salt reduction strategy. These interventions were delivered through several ways, including in-person education [42, 55, 60], group education [31, 59], a combination of individual and group sessions [40], web-based programs [46], written educational materials such as booklets [50], a combination of group sessions and written educational materials [49, 52], a combination of individual and group counselling, motivational campaign, written educational materials and audio-visual aids [41], salt-reduced cooking class [38], a combination of the public lecture, face-to-face sessions, and promotional materials [21], health education lessons of the schools [28, 56], app-based education program [61], transmitting messages through children and delivering educational materials in the form of a newsletter [28], and combination of the health education lessons and gardening club of the school [56]. There were some differences between interventions in terms of the frequency of the education sessions and lengths of the follow-up period. The intervention duration varied between two weeks to 24 months. Li et al. reported that community-based multi-component health education program reduced salt intake but did not significantly affect BP [21]. Since, the baseline data of this study were not reported, it was not included in the current meta-analysis. The remaining 20 studies, including data from 5,703 participants, were entered into the meta-analysis. Ten studies reported a significantly greater reduction in SBP than the control group [31, 40, 41, 46, 50, 59–61], six reported non-significant improvement [28, 38, 52, 55], three studies indicated a significantly high increase in SBP than the control group [49, 56], and one study showed non-significant increase than the control group [42]. The pooled results indicated that health education interventions resulted in a significant reduction in SBP (WMD: -2.75 mmHg, 95% CI: -4.30, -1.19, P = 0.001) (Fig 2A). However, there was a substantial heterogeneity in the studies (I^2^ = 94.8%, P < 0.001). In terms of DBP, ten trials reported a significantly greater decrease in DBP compared to the control group [31, 40–42, 59–62], seven showed non-significant improvements [28, 38, 46, 52, 55], two trials reported a significant increase [56], and one reported a non-significant increase [49]. Based on the pooled results, health education interventions significantly reduced DBP (WMD: -2.11 mmHg, 95% CI: -3.17, -1.06, P < 0.001). The heterogeneity levels were considerably high (I^2^ = 92.6%, P < 0.001) (Fig 2B).

Subgroup analysis showed that nutritional education led to a greater reduction in SBP among individuals aged >60 years (WMD: -5.94, CI: -7.22, -4.60, P < 0.001), and in the subset of studies with intervention duration of >6.6 months (WMD: -4.49, CI: -6.56, -2.42, P < 0.001) (Table 3). Moreover, the net change in SBP was significant only in high and medium-quality studies. No significant differences were detected for SBP when groups were separated by sample size and participants’ HTN status. Accordingly, the subgrouping revealed that nutritional education resulted in a higher reduction in DBP in participants aged >60 years (WMD: -4.67, CI: -6.14, -3.20, P < 0.001), and also in sample size >290 (WMD: -3.21, CI: -4.47, -1.95, P<0.001). The pooled effect size in DBP did not significantly differ when groups were separated by intervention duration and participants’ HTN status. Moreover, the I^2^ statistic was reduced to <50% for SBP when the analysis was restricted to high-quality trials, groups with age >60 years, and trials with intervention duration >6.6 months, and for DBP when high-quality studies and studies with intervention duration <2 months were analyzed.

**Table 3:** Results of subgroup analyses according to study or participant characteristics.

	No. of studies	WMD (95% CI)	P _within group_	P _between group__*_	P_heterogeneity_	I^2^, %	No. of studies	WMD (95% CI)	P _within group_	P _between group__*_	P_heterogeneity_	I^2^, %
		SBP						DBP				
**Education**												
Total	20	-2.75 (-4.30, -1.19)	0.001		<0.001	94.8	20	-2.11 (-3.17, -1.06)	<0.001		<0.001	92.6
***Quality***				0.40						0.41		
heigh	7	-1.72 (-2.51, -0.92)	<0.001		0.312	15.5	7	-1.25 (-1.74, -0.76)	<0.001		0.655	0.0
medium	11	-2.77 (-4.66, -0.87)	0.004		<0.001	95.1	11	-2.25 (-3.78, -0.72)	0.004		<0.001	94.7
low	2	-2.74 (-4.31, -1.19)	0.150		0.027	79.4	2	-2.63 (-6.31, 1.06)	0.162		0.146	52.7
***Age***				<0.001						<0.001		
<25	6	-0.03 (-3.65, 3.58)	0.985		<0.001	97.0	6	0.37 (-2.02, 2.76)	0.761		<0.001	94.0
25–60	6	-2.29 (-4.33, -0.25)	0.028		<0.001	82.0	6	-2.31 (-4.18, -0.44)	0.016		<0.001	91.4
>60	7	-5.94 (-7.22, -4.6)	<0.001		0.382	6.0	7	-4.67 (-6.14, -3.20)	<0.001		0.056	51.2
***Hypertension status***				0.890						0.250		
Normotensives	0	-	-		-	-	0	-	-		-	-
Hypertensives	7	-2.97 (-6.75, 0.81)	0.124		<0.001	94.6	7	-2.92 (-4.48, -1.35)	<0.001		<0.001	78.2
Mix of hypertensives and normotensives	13	-2.63 (-4.67, -0.59)	0.012		<0.001	94.8	13	-1.67 (-3.06, -0.29)	0.018		<0.001	94.7
***Duration***				0.160						0.08		
<2 months	5	-3.44 (-7.91–1.04)	0.132		0.001	79.0	5	-4.21 (-5.88, -2.54)	<0.001		0.133	43.3
2–6.6 months	9	-1.20 (-3.88,1.49)	0.382		<0.001	90.9	9	-0.65 (-3.39, 2.08)	0.640		<0.001	95.6
>6.6months	6	-4.49 (-6.56, -2.42)	<0.001		0.304	96.6	6	-2.70 (-3.89, -1.51)	<0.001		<0.001	91.7
***Sample size***				0.330						0.14		
<290	13	-2.04 (-4.69, 0.61)	0.131		<0.001	93.9	13	-1.39 (-3.42, 0.64)	0.180		<0.001	93.0
>290	7	-3.87 (-6.29, -1.44)	0.002		<0.001	96.1	7	-3.21 (-4.47, -1.95)	<0.001		<0.001	92.8
**Self-help materials**												
Total	10	-1.36 (-3.46, 0.74)	0.205		0.003	64	6	-1.15 (-2.46, 0.16)	0.085		0.011	59.6
***Quality***				0.93						0.60		
heigh	5	-1.61 (-3.78, 0.56)	0.147		0.228	29.1	4	-1.01 (-2.15, 0.13)	0.082		0.601	0
medium	5	-1.38 (-4.95, 2.19)	0.448		0.012	72.1	5	-1.76 (-4.33, 0.79)	0.177		0.002	76.7
low												
***Age***				<0.001						0.01		
<25	1	2.60 (0.92, 4.28)	0.002		-	-	1	1.40 (-0.09, 2.89)	0.067		-	-
25–60	6	-2.60 (-4.51, -0.68)	0.008		0.341	11.7	6	-1.55 (-2.97, -0.13)	0.032		0.123	42.3
>60	3	-0.83 (-3.51, 1.84)	0.542		0.418	0.0	2	-1.52 (-3.89, 0.85)	0.208		0.260	21.1
***Hypertension status***				0.10						<0.001		
Normotensives	2	0.43 (-4.43, 5.28)	0.863		0.022	81.0	2	0.99 (-0.30, 2.25)	0.123		0.321	0
Hypertensives	4	-3.87 (-6.40, -1.34)	0.003		0.419	0	4	-2.91 (-4.33, -1.49)	<0.001		0.517	0
Mix of hypertensives and normotensives	4	-0.70 (-2.78, 1.38)	0.511		0.557	0	3	-0.45 (-1.87, 0.98)	0.537		0.899	0
***Duration***				-						-		
<2 months	10	-1.36 (-3.46, 0.744)	0.205		0.003	64	9	-1.15 (-2.46, 0.16)	0.085		0.011	59.6
2–6.6 months	0	-	-		-	-	0	-	-		-	-
>6.6months	0	-	-		-	-	0	-	-		-	-
***Sample size***	0			-			0			-		
<290	10	-1.36 (-3.46, 0.74)	0.205		0.003	64	6	-1.15 (-2.46, 0.16)	0.085		0.011	59.6
>290	0	-	-		-	-	0	-	-		-	-
**Salt substitute**												
Total	14	-7.44 (-8.82, -6.06)	<0.001		<0.001	93.3	14	-3.77 (-5.21, -2.32)	<0.001		<0.001	97.2
***Quality***				0.700						0.391		
heigh	7	-7.80 (-9.84, -5.77)	<0.001		0.006	66.5	7	-4.28 (-6.05, -2.50)	<0.001		<0.001	76.2
medium	7	-7.11 (-8.97, -5.25)	<0.001		<0.001	96.3	7	-3.27 (-5.31, -1.24)	<0.001		<0.001	98.6
low	-						-					
***Age***				0.150						0.010		
<25	1	-7.14 (-7.71, -6.57)	<0.001		-	-	1	-5.12 (-5.66, -4.58)	<0.001		-	-
25–60	5	-9.25 (-11.35, -7.15)	<0.001		0.584	0	5	-5.56 (-7.10, -4.02)	<0.001		0.677	0
>60	7	-7.09 (-9.57, -4.60)	<0.001		<0.001	90.7	6	-2.56 (-4.12, -1.01)	0.001		<0.001	88.8
***Hypertension status***				0.270						0.633		
Normotensives	1	-7.00 (-8.73, -5.27)	<0.001		-	-	1	-4.60 (-6.52, -2.68)	<0.001		-	-
Hypertensives	10	-8.16 (-9.71, -6.62)	<0.001		0.003	64.6	10	-4.24 (-5.61, -2.87)	<0.001		<0.001	83.4
Mix of hypertensives and normotensives	3	-5.65 (-8.38, -2.91)	<0.001		<0.001	97.9	3	-3.04 (-6.13, -0.04)	0.053		<0.001	99.3
***Duration***				0.830						0.052		
<2 months	3	-8.19 (-10.80, -5.58)	<0.001		0.738	0	3	-6.26 (-8.43, -4.08)	<0.001		0.467	0
2–6.6 months	6	-7.79 (-10.03, -5.55)	<0.001		0.002	74.2	6	-2.95 (-4.67, -1.23)	0.001		<0.001	80.4
>6.6months	5	-6.84 (-8.93, -4.74)	<0.001		<0.001	97.4	5	-3.85 (-6.27, -1.44)	0.002		<0.001	99.00
***Sample size***				0.010						0.140		
<290	10	-8.37 (-9.67, -7.08)	<0.001		0.020	54.1	10	-4.28 (-5.48, -3.07)	<0.001		<0.001	72.1
>290	4	-5.37 (-7.67, -3.06)	<0.001		<0.001	96.9	4	-2.56 (-5.13, -0.01)	0.051		<0.001	98.9

CI: Confidence interval, SBP: Systolic blood pressure, DBP: diastolic blood pressure, WMD: Weighted mean difference

*Between groups comparisons are obtained from inverse variance method

### Effect of self-help materials on SBP and DBP

Ten studies used self-help materials as a salt reduction strategy. Five of these trials applied a self-monitoring urinary salt excretion measurement device to motivate individuals to avoid high salt intake [34–37, 39]. Morikawa et al. reported a significantly greater reduction only in DBP in the intervention group compared to the control group [39]; in addition, Yasutake et al. found that the control group had a significantly greater reduction in SBP compared to the intervention group [34]. Additionally, three studies found no significant difference between intervention and control groups [35–37].

Three trials evaluated the effectiveness of smartphone applications that allowed individuals to identify lower-salt options [47, 48, 51]. Dorsch et al. found that the intervention group had a significantly greater reduction in SBP compared to the control group [47]. Payne Riches et al. and Eyles et al. reported no significant difference between the two groups in terms of SBP and DBP and only SBP, respectively [48, 51]. DBP was not measured at the end of study by Eyles et al. [51]. In the study by Pinjuh Markota et al., the intervention group received warning stickers to be placed on all home salt containers [53]. Results indicated that the intervention group had a significantly greater reduction in both SBP and DBP compared to the control group. The meta‐analysis of all studies including data from 972 participants, which evaluated the effect of using self-help materials, showed no differences in SBP (WMD: -1.36 mmHg, 95% CI: -3.46, 0.74, P = 0.205; I^2^ = 64%, P = 0.003), and DBP (WMD: -1.15 mmHg, 95% CI: -2.46, 0.16, P = 0.085; I^2^ = 59.6%, P = 0.011) between the intervention and control groups (Fig 2A and 2B).

Subgroup analysis revealed that using self-help materials was more effective in lowering BP among participants aged between 25–60 years (WMD: -2.60 mmHg, CI: -4.51, -0.68, P = 0.008 and WMD: -1.55 mmHg, CI: -2.97, -0.13, P = 0.032 for SBP and DBP, respectively) (Table 3). Furthermore, the pooled effect size of the interventions was greater in the hypertensive groups (WMD: -3.87 mmHg, CI: -6.40, -1.34, P = 0.003 and WMD: -2.91 mmHg, CI: -4.33, -1.49, P<0.001 for SBP and DBP, respectively). The results did not indicate any significant effect of using self-help materials on SBP and DBP when subgrouping was carried out according to study quality. In addition, the heterogeneity level was reduced in subgrouping according to the HTN status, age category, and study quality.

### Effect of salt substitutes on SBP and DBP

Seventeen trials examined the effects of various salt substitutes on BP improvement. The majority of trials used a mixture of varying amounts of NaCl, KCl, MgSO2, and Mg4K(NH4)3Cl12·24H2O] in combination with other micronutrients (e.g., calcium, folic acid) as salt substitutes and two trials evaluated the effect of substituting NaCl with the combination of NaCl and 3% chitosan. The China Salt Substitute Study showed that replacing salt with low-sodium alternatives in high-risk rural northern Chinese adults can lead to a significant decrease in SBP [23]. However, this study was not included in our meta-analysis because it did not report the end of the follow-up data. Recently, in a study by Bernabe-Ortiz et al., replacing regular salt with potassium-enriched substitutes resulted in an average reduction of 1.23 mm Hg (P = 0.004) in SBP and 0.72 mm Hg (P = 0.022) in DBP, after adjusting for clustering and time effects. As a stepped-wedge cluster randomized trial design was used in this study, it was also not included in the meta-analysis [25]. The remaining 14 studies, including data from 23,587 participants were entered in this meta-analysis. The use of salt substitutes in all the included trials showed a significant reduction in SBP compared to the control groups. The pooled results indicated that the salt substitute interventions resulted in a significant reduction in SBP (WMD: -7.44 mmHg, 95% CI: -8.82, -6.06, P < 0.001; I^2^ = 93.3%, P < 0.001) (Fig 2A). Regarding DBP, 11 trials reported a significantly greater decrease in DBP compared to the control groups, while two studies reported a non-significant improvement. The pooled data showed a beneficial effect of salt substitutes on DBP (WMD: -3.77 mmHg, 95% CI: -5.21, -2.32, P < 0.001; I^2^ = 97.2%, P < 0.001) (Fig 2B).

Subgroup analysis indicated greater numerical DBP reduction in groups aged 25–60 years (WMD: -5.56 mmHg, CI: (-7.10, -4.02, P = 0.010) (Table 3). The results did not suggest any different effect of salt substitution on DBP when subgrouping was performed according to hypertension status, intervention duration, study quality, and sample size. Considering DBP, the effect size of the intervention did not differ significantly in any subset of studies. Moreover, subgroup analyses revealed that the heterogeneity level reduced in subgrouping according to age category, and intervention duration.

### Effect of food reformulation on SBP and DBP

Two cross-over trials examined the effect of food reformulation in terms of salt content as a measure to help achieve a reduction in salt intake and BP. In a study by Ferrante et al., replacing ordinary bread containing 2% salt with low-salt bread containing 1.4% salt resulted in a significant reduction in SBP (1.66 mmHg, P = 0.005) and DBP (0.76 mmHg, P = 0.029) during the low-salt bread intake period compared to the usual-salt period [22]. Cashman et al. examined the effect of replacing bread and a few other foods with equivalent low-salt ones [24]. The results showed significantly lower SBP (by 3.3 mmHg on average; P < 0.0001) during the salt restriction period compared to the usual dietary period, but no significant difference was found for DBP. However, the results of these studies were presented based on the paired comparison. So, they were not included in the current meta-analysis.

### Meta-regression analysis

A meta-regression was performed to find a potential source of heterogeneity and also to assess the association between the effect sizes of each type of salt reduction intervention with potential confounders (see S2 Table). The results showed a significant correlation between age of participants and the effect size for nutrition education on SBP and DBP (ß = -2.64, P = 0.016 and ß = -2.35, P = 0.013, respectively). There was no significant association between study quality, age group, HTN status, intervention duration, and sample size with the effect size of salt substitution and using self-help materials.

### Publication bias

**Fig 3:**
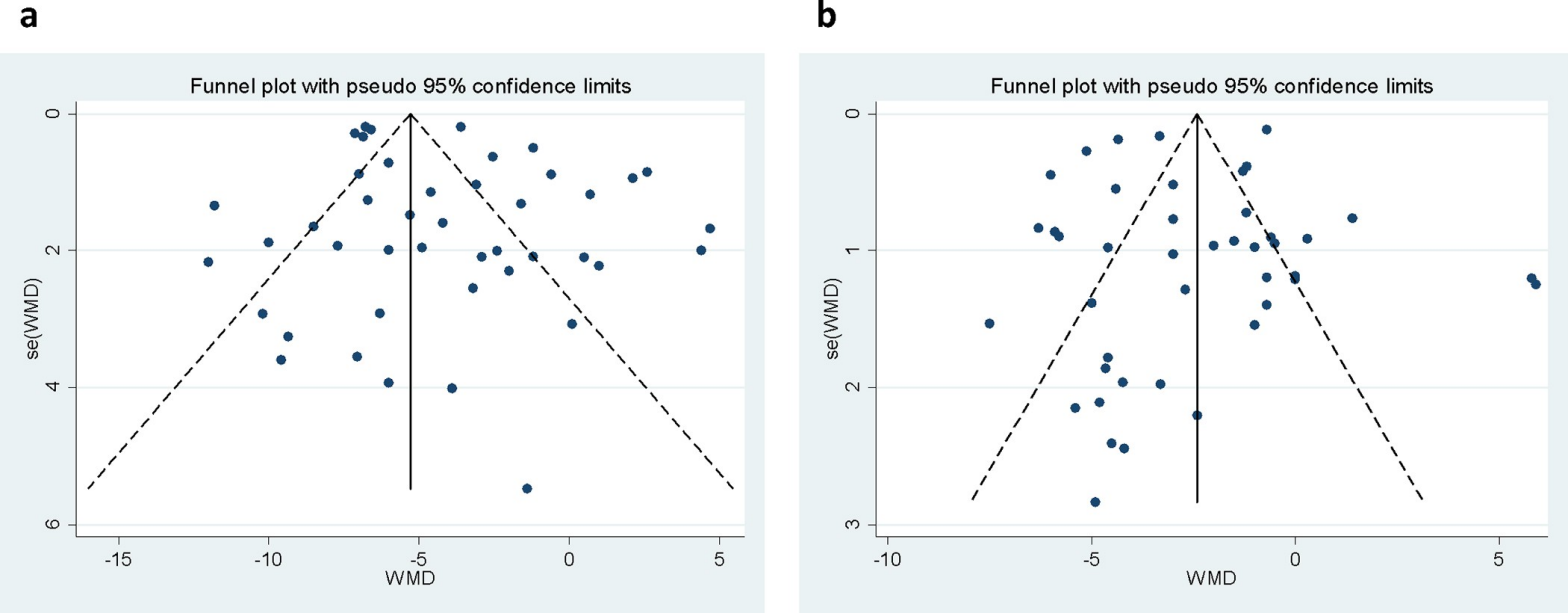
Funnel plots of the included studies in the meta-analysis of the effect of salt reduction intervention on SBP (a) and DBP (b).

The visual inspection of funnel plots revealed slight evidence of the asymmetric distribution among studies that examined the effect of all salt reduction strategies on SBP (Fig 3A). However, the results from Egger’s test (P = 0.092) and Begg’s rank correlation test (P = 0.716) did not show any evidence of publication bias. Based on the results of the trim and fill method, there were no missing trials for SBP. In addition, there was no evidence of publication bias based on visual inspection of funnel plots (Fig 3B), Egger’s test (P = 0.618), and Begg’s test (P = 0.975) for DBP.

## Discussion

This systematic review and meta-analysis evaluated the effect of different salt reduction strategies on BP. In the current review, different studies with various interventions, diverse components, and different target populations were included. Of 44 studies meeting the inclusion criteria of the meta-analysis, 20 trials were conducted using nutrition education strategies, ten studies used self-help materials, and 14 trials employed salt substitutes. The results showed significant effects of overall salt reduction intervention on SBP and DBP levels. The positive effect of salt reduction on BP and the risk of CVD has been clearly demonstrated in numerous studies [63]. Although, different potential mechanisms have been revealed, the exact underlying mechanism for BP reduction due to salt limitation is unclear. Increased salt consumption may provoke water retention, leading to an increase in blood volume and peripheral vascular resistance; thus, salt restriction could weaken these effects and reduce BP [64]. Furthermore, it has been suggested that salt reduction could prevent the production of reactive oxygen species (ROS), decrease nitric oxide activity, and lower BP [65].

According to the WHO recommendations, reducing dietary salt intake is one of the most cost-effective healthcare interventions to reduce BP and prevent non-communicable diseases such as CVD at the population level [66]. Dietary salt-lowering programs have successfully been launched in several countries. For example, Finland developed a comprehensive approach to reduce salt intake in the late 1970s; this program consisted of public awareness campaigns, warning or better choice labels on high-salt foods, cooperation with the food industry, and legislative restrictions on the maximum salt content of some groups of products. The mean daily salt intake in Finland decreased from approximately 14.5 g in men (not measured in women) to about 11 g in men and 7 g in women from the 1970s to the late 1990s [67]. In addition, a one-third decrease in salt consumption was accompanied by a reduction of an average of 10 mm Hg or more in both SDP and DBP during the 30 years [68]. The UK, Australia, Ireland, and several other countries have also successfully launched salt reduction programs in collaboration with the food industry [69–71]. Moreover, the effectiveness of various salt redaction interventions has been examined in numerous studies. Recently, Jin et al. conducted a meta-analysis study on eight high-quality RCTs developed in China to evaluate the effect of different salt reduction strategies on BP improvement [14]. Our results were consistent with the results of this study, which showed that salt substitutes could lead to a considerable reduction in SBP (-5.67 mmHg) and DBP (-1.95 mmHg) among Chinese adults.

### Health education

The current meta-analysis showed that nutrition education is an effective approach in achieving reductions in both SBP and DBP. Overall, the quality of the study and the mean age of participants could be the potential sources of high heterogeneity for SBP, and only the mean age of participants for DBP. In addition, educational approaches employed in trials were diverse in terms of delivery settings (workplaces, schools, communities, etc.), trained components of education programs, and the ways they were delivered (group training sessions, print-materials, web-based tools, etc.). Therefore, they could be a potential source of heterogeneity. Also, some studies had a low quality based on the JBI checklist, and one of the studies had a non-RCT design. Therefore, they could cause potential biases in the pooled estimates, so the results of subgroup analysis revealed that the effect of interventions in high and medium-quality trials was significant, but lower than the pooled data. Based on subgroup analysis, nutrition education in participants with a mean age of >60 years had a more reducing effect on SBP and DBP, and in studies with longer follow-up duration had a more reducing effect only on SBP. In a recent meta-analysis by Jin et al., the pooled results of trials showed that health education programs significantly reduced SBP and DBP (-8.1 mmHg in SBP and -4.5 mmHg in DBP) [14]. Our results were in line with this study which indicated that high-quality trials resulted in small effect size. Recently, several systematic reviews and meta-analyses have demonstrated the beneficial role of self-management and lifestyle education programs in BP control [72–74]. For example, Tam et al. showed that educational interventions for lifestyle modification had a moderate effect in improving SBP and DBP [72].

The effectiveness of public education as a key component of salt reduction strategies has also been proven in population-wide interventions [71, 75–77]. Health education efforts in the UK via salt reduction campaigns using various media outlets have led to increased public awareness and reduction in salt intake [76, 78]. Successful education strategies can lead to changes in social norms related to salt intake, and consequently health-related behavior such as increased demand for healthier and lower-salt products. Moreover, increased consumer awareness through education can encourage the food industry to make recipe reformulations and reduce salt content. In contrast, some experiences revealed that education or information alone, without additional economic or environmental changes, is generally insufficient to make sustainable results regarding behavior change [79, 80]. It seems that integrated multicomponent approaches that combine both nutrition education and public awareness efforts with food-environment change interventions were more effective in improving salt reduction behaviors, and ultimately cardiovascular outcomes.

Interestingly, our findings showed a noticeable effect of nutritional education in salt intake reduction on SBP and DBP among participants with a mean age of >60. However, it should be noted that random allocation and concealment were inappropriate in four of six trials [40, 50, 55, 60]. Therefore, they could cause potential biases in the pooled estimates. Moreover, a previous meta-analysis of RCTs indicated that a decline in BP with salt reduction was greater in older people than younger ones [9]. Furthermore, other uncontrolled factors, such as therapeutic adherence and consideration of who cooked at home, could contribute to BP improvement. In agreement with our findings, several studies suggested that health education interventions such as physical fitness, weight control, and lifestyle modification would improve health complications and biomedical functions in the elderly population [81–83]. Figar et al. showed that educational intervention based on a patient empowerment model in elderly patients led to an increase in BP control and reduction in BP in the analysis of 24-h ambulatory BP monitoring (ABPM) [84]. Furthermore, Ozoemena et al. revealed that a community-based health education program designed for older adults increased HTN knowledge and improved prevention and self-care behaviors [83].

### Self-help materials

Our meta-analysis of ten studies on the use of self-help materials showed no significant pooled effect size in both SBP and DBP. Additionally, the pooled effect of five of these trials that used self-monitoring urinary salt excretion measurement devices was not significant, based on subgroup analysis (the result was not presented). According to previous reports, there was no considerable reduction in the daily salt intake in salt-conscious patients who were alert to reduce their daily salt intake, suggesting the importance of repeated monitoring of salt intake by urinary sodium excretion and providing feedback to the patients as a practical way to encouraging the achievement of salt restriction in hypertensive individual [85, 86]. Furthermore, this method seems useful for the appropriate management of other salt reduction strategies and judgment of whether the goal of salt intake restriction has been achieved [87]. The reliable method for this purpose is the measurement of the sodium extraction in 24-h pooled urine; however, repeated implementation of 24-h urinary tests is not feasible. Finally, a simple self-monitoring device can be used as a salt-reduction tool to provide a repeated measurement of urinary salt excretion at home [88]. However, this strategy’s effectiveness for both salt intake and BP reduction has been shown only in before-after design studies with weak evidence [89, 90]. Besides, several studies have indicated the controversial effects of self-monitoring of blood glucose in patients with type 2 diabetes [91, 92]. Accordingly, these diverging results suggest that if self-monitoring of sodium extraction is accompanied with other salt reduction strategies, especially nutrition education, it might be effective in salt intake reduction and BP improvement. It is worth mentioning that the follow-up duration of the included studies in this meta-analysis was less than two months; hence, no significant change in BP might be due to the short trial period. Altogether, further studies with a longer period, larger sample size, and considering the effect of other factors such as physical activity and energy intake are needed to confirm these results.

Three studies in the current meta-analysis evaluated the effectiveness of smartphone application on BP reduction in patients with diagnosed CVD and patients with elevated blood pressure [47, 48, 51]. SaltSwitch app enables shoppers to scan the barcode of packaged food with a smartphone to receive simple health information to make a low-salt food choice. The results revealed no significant difference in SBP and DBP between the two groups. In the study by Dorsch et al., using LowSalt4Life, a just-in-time adaptive mobile app intervention that recommends lower dietary sodium food alternatives at home, restaurants, and grocery stores, resulted in a significant reduction in SBP and DBP [47]. Limited studies are available regarding the efficacy of mobile health (mHealth) interventions in improving self-management of BP. A review of the impact of digital health interventions on CVD risk factors showed that compared with usual care, digital interventions significantly reduced CVD outcomes, weight, body mass index (BMI), and SBP among primary prevention trials, but no reduction in SBP in secondary prevention digital health interventions trials was found [93]. However, given that there is no robust study about the efficacy of smartphone applications on BP management, long-term RCTs in various age groups are required to evaluate their usefulness. Another study included in the present meta-analysis assessed the impact of placing warning stickers about the adverse effects of excessive salt on household salt containers [53]; a significant reduction was found in SBP and DBP in the intervention group [53]. The positive impact of warning messages on tobacco packages on communicating the risk of smoking has also been demonstrated in the previous literature [94, 95]. Similarly, a recent meta-analysis revealed that placing a warning label on food and alcohol products has an enormous impact on selecting unhealthy products [96]. Nutrient warning labels may offer some advantages, such as being low-cost, clarity of message, and exposure to all family members. It seems that long-term interventions might be useful to reduce salt intake and BP, especially in developing countries where salt intake is mainly derived from adding salt at the table or during home cooking procedures [97, 98].

Although the effect of using self-help material on SBP and DBP was not significant, subgrouping showed significant results for the trials performed among hypertensive individuals and those aged 25–60 years. Previous research has mentioned that older people have difficulty remembering how to use new technologies [99]. Although such self-help devices are helpful for a healthy life, in comparison with younger people, older individuals do not show much interest in adopting new technologies in healthcare activities, communication, and customer service [100]. Younger individuals, who are more likely to suffer from undiagnosed and untreated hypertension [101] are more eager to use digital devices and self-help materials. For better results from self-help materials, it is necessary to combine their development with education on reducing the total amount of salt used.

### Salt substitutes

Our results indicated that the salt substitute interventions resulted in a significant reduction in SBP and DBP; these interventions had the highest effect size among various types of salt reduction strategies evaluated in the current meta-analysis. Furthermore, a significant decrease in mean SBP and DBP values was detected in all age groups, follow-up durations, baseline HTN status, and sample size. A moderate to high heterogeneity was found between the included trials. Age category, and intervention duration were the potential sources of heterogeneity based on subgroup analysis. Recently, Bernabe-Ortiz et al. evaluated the effect of replacing regular salt with potassium-enriched substitutes at the population level, using a stepped-wedge cluster randomized trial design [25]. The results support the effectiveness of population-wide salt-substitution strategy on BP and HTN incidence. Besides BP reduction, replacing regular salt with low-sodium alternatives yielded additional health benefits, such as a direct effect on reducing the risk of stroke, kidney stones, and osteoporosis [102, 103]. Moreover, in a salt-substitution trial conducted in Taiwan, the risk of CVD mortality was 41% less in those assigned to the potassium-enriched salt substitute group at 31 months (age-adjusted hazard ratio, 0.59 [95% CI, 0.37–0.95]) [104]. In previous meta-analyses, a significant positive effect of salt substitutes on SBP and DBP was reported [105, 106]; however, in the present study, we included a larger number of related studies so as to conduct a stratified analysis on different factors, including age, baseline HTN status, follow-up duration, and sample size, which can influence the effect of the salt substitute on BP.

The anti-hypertensive effect of salt substitutes is exerted not only by the decline in sodium intake but also due to the increase in potassium, magnesium, and calcium intake or other edible substances such as chitosan. It has been established that potassium has a favorable impact on BP control [107, 108]. Most trials about salt substitutes have used potassium-enriched salt as an appropriate agent for food salting that has the saltiness of regular salt but with anti-hypertensive effects. Several mechanisms for the hypotensive effect of potassium have been proposed, including the development of a negative sodium balance due to the downregulation of NaCl cotransporter, decreased renal renin release, and enhanced urine flow [108].

A major concern in the salt substitution implementation strategy is its acceptability caused by an alteration in flavor and palatability of salt because of the replacement of sodium with potassium [109]. To overcome these problems, a limited percentage of KCl has been used in combination with other nutritionally accepted agents (MgCl2, MgSo2, etc.). In studies that explored the taste acceptability of six different potassium-enriched salt substitutes, more than 80% of individuals did not differentiate between regular salt and potassium-enriched salt substitutes that contain less than 30% KCl [110]. The other concern related to potassium-enriched salt substitutes is a possible increased risk of hyperkalemia, consequent arrhythmias, and sudden cardiac death, especially among individuals suffering from impaired potassium excretion, such as chronic kidney disease [111]. However, there is considerably weak evidence regarding the relationship of potassium-enriched salt with serum potassium levels and the occurrence of hyperkalemia in patients with chronic kidney disease and others at risk for hyperkalemia [112]. It is important to note that most trials about the salt substitutes’ effectiveness excluded the population at risk for hyperkalemia, such as people with decreased kidney function or elevated potassium levels at baseline. The China Salt Substitute and Stroke Study (SSaSS), involving 20,995 persons with a history of stroke or age ≥ 60 years with high blood pressure, clinical hyperkalemia, and sudden death was assessed as a key indicator of safety [33]. The results indicated that the rate of serious adverse events attributed to hyperkalemia was not significantly higher with the salt substitute (75% sodium chloride and 25% potassium chloride) than with regular salt. Also, there was no evidence of significant increase in the risk of sudden death that might be caused by hyperkalemia-induced arrhythmic events. Large clinical studies are needed to evaluate the adverse effects of salt substitutes, especially among populations with a high prevalence of hyperkalemia. Taken together, dietary salt substitution intervention has remarkable benefits to public health by having easy access, low-cost [113], and fewer side effects, and is an effective strategy for modifying the social and financial burden of HTN and CVD.

Several systematic reviews have demonstrated an increase in the number of countries applying salt reduction approaches since 2010 [114, 115]. Understanding the population’s main dietary sodium source is fundamental to help to choose the best salt reduction strategies in any community setting [116]. In many high-income countries where the majority of salt in the diet comes from processed or packaged foods, food product reformulation, front-of-pack labelling schemes, and interventions in settings might bring more positive changes in salt reduction [117, 118]. However, in countries where the primary source of salt is discretionary salt added by the individual during food preparation or at the table—a trend that is common in many low- and middle-income countries—interventions that change people’s salt-related behavior such as consumer education and replacing salt with salt substitutes might provide more viable approach [115, 117].

It is worth mentioning that, given the limited public resources available to meet government priorities and objectives, identifying the most appropriate policies should be done by considering all attributes of the interventions and multiple criteria for assessing them (e.g., effectiveness, implementation costs, feasibility, sustainability, etc.) in accordance with the socioeconomic, cultural, and technological status, along with consumption patterns in each community [119–121].

### Limitations and strength

As far as the researchers of the current study investigated, this study is the first comprehensive systematic review and meta-analysis evaluating the effectiveness of various types of nutrition intervention strategies on the improvement of BP, regardless of age, sex, and health conditions of participants. Additionally, the large number of studies included in this meta-analysis allowed for subgroup analysis, which strengthened the statistical power of the research. However, several limitations of this meta-analysis should also be mentioned. Firstly, only three types of salt reduction interventions were included in the meta-analysis. Other types of interventions such as taxation, food labelling, and food reformulation were reported in few studies. Secondly, we could not completely compare the effectiveness of the interventions between hypertensive and normotensive individuals. Only three trials of this meta-analysis were among normotensive individuals and the remaining ones were conducted on hypertensive or mixed hypertensive and normotensive individuals. Thirdly, we were unable to perform subgroup analysis based on gender or BMI because of insufficient data for these two categories. Previous studies have shown that BP responses to lowering dietary salt were more prominent in persons with obesity [122], and also in women than in men may be due to the interactions between sex hormones and renal function [123, 124]. These findings warrant future research to investigate salt reduction interventions in genders and BMI groups. Additionally, numerous epidemiological and experimental studies have indicated the critical role of dietary patterns and physical activity in HTN prevention and management [124–126]. However, the majority of the studies in our systematic review and meta-analysis overlooked the importance of these confounding factors. Furthermore, most of the included studies did not provide any information about using anti-hypertensive drugs or drugs affecting BP during the intervention. Adjusting for the mentioned confounding factors could have altered the results and conclusions of some studies. In addition, there was substantial heterogeneity among studies even in several different sub-groups, which might stem from the inconsistencies in the study’s design, sample source, intervention methods, participants’ genetic background, and dietary habits.

## Conclusions

In conclusion, the results of the present meta-analysis indicated that salt substitution and nutrition education are effective strategies for lowering SBP and DBP. Further large and well-designed studies are needed to clarify the efficacy of using self-help materials in BP improvement. It seems that high-quality trials in populations with different socioeconomic and cultural status, genetic backgrounds, and health status are needed to achieve efficient and sustainable interventions.

## Supporting information

S1 Checklist PRISMA checklist. (DOCX)

S1 Table The detailed search strategy in different databases. (DOCX)

S2 Table Meta-regression on the association of intervention effect on SBP and DBP change with study quality, age category, hypertension status, study duration, and sample size. (DOCX)
