# Supplementary material for: Effect of salt reduction interventions in lowering blood pressure: A comprehensive systematic review and meta-analysis of controlled clinical trials
Source: PLoS One. 2022 Dec 7;17(12):e0277929. doi: 10.1371/journal.pone.0277929 (PMC9728935; doi:10.1371/journal.pone.0277929)
Supplement: S1 Table — (DOCX) [file pone.0277929.s002.docx]

| **Table S1. Search strategies and the number of records according to different electronic database.** | | |
| --- | --- | --- |
| **Database** | **Search strategy** | **Number of records** |
| Pubmed | (((((salt[Title/Abstract]) OR (sodium[Title/Abstract])) AND (((intake[Title/Abstract]) OR (consum*[Title/Abstract])) OR (diet[Title/Abstract]))) AND ((((((((reduc*[Title/Abstract]) OR (restrict*[Title/Abstract])) OR (minimi*[Title/Abstract])) OR (eliminat*[Title/Abstract])) OR (limit[Title/Abstract])) OR (low[Title/Abstract])) OR (free[Title/Abstract])) OR (curb[Title/Abstract]))) AND (((blood pressure[Title/Abstract]) OR (hypertension[MeSH Terms])) OR (hypertension[Title/Abstract]))) AND ((((((((polic*[Title/Abstract]) OR (intervention[Title/Abstract])) OR (initiative[Title/Abstract])) OR (regulation[Title/Abstract])) OR (action[Title/Abstract])) OR (strateg*[Title/Abstract])) OR (program[Title/Abstract])) OR (activit*[Title/Abstract])) | 2812 |
| Scopus | TITLE-ABS-KEY(salt) OR TITLE-ABS-KEY(sodium) AND TITLE-ABS-KEY(blood pressure) OR TITLE-ABS-KEY(hypertension) AND TITLE-ABS-KEY(intake) OR TITLE-ABS-KEY(consum*) OR TITLE-ABS-KEY(diet) AND TITLE-ABS-KEY(reduc*) OR TITLE-ABS-KEY(restrict*) OR TITLE-ABS-KEY(minimi*) OR TITLE-ABS-KEY(eliminat*) OR TITLE-ABS-KEY(limit) OR TITLE-ABS-KEY(low) OR TITLE-ABS-KEY(free) OR TITLE-ABS-KEY(curb) AND TITLE-ABS-KEY(polic*) OR TITLE-ABS-KEY(intervention) OR TITLE-ABS-KEY(initiative) OR TITLE-ABS-KEY(regulation) OR TITLE-ABS-KEY(action) OR TITLE-ABS-KEY(strateg*) OR TITLE-ABS-KEY(program) OR TITLE-ABS-KEY(activit*) AND ( LIMIT-TO ( PUBYEAR,2022) OR LIMIT-TO ( PUBYEAR,2021) OR LIMIT-TO ( PUBYEAR,2020) OR LIMIT-TO ( PUBYEAR,2019) OR LIMIT-TO ( PUBYEAR,2018) OR LIMIT-TO ( PUBYEAR,2017) OR LIMIT-TO ( PUBYEAR,2016) OR LIMIT-TO ( PUBYEAR,2015) OR LIMIT-TO ( PUBYEAR,2014) OR LIMIT-TO ( PUBYEAR,2013) OR LIMIT-TO ( PUBYEAR,2012) OR LIMIT-TO ( PUBYEAR,2011) OR LIMIT-TO ( PUBYEAR,2010) OR LIMIT-TO ( PUBYEAR,2009) OR LIMIT-TO ( PUBYEAR,2008) OR LIMIT-TO ( PUBYEAR,2007) OR LIMIT-TO ( PUBYEAR,2006) OR LIMIT-TO ( PUBYEAR,2003) OR LIMIT-TO ( PUBYEAR,2002) OR LIMIT-TO ( PUBYEAR,2001) OR LIMIT-TO ( PUBYEAR,2000) ) | 7808 |
| Embase | (salt:ab,ti OR sodium:ab,ti) AND (intake:ab,ti OR consum*:ab,ti OR diet:ab,ti) AND (reduc*:ab,ti OR restrict*:ab,ti OR minimi*:ab,ti OR eliminat*:ab,ti OR limit:ab,ti OR low:ab,ti OR free:ab,ti OR curb:ab,ti) AND ('blood pressure':ab,ti OR hypertension:ab,ti) AND (polic*:ab,ti OR intervention:ab,ti OR initiative:ab,ti OR regulation:ab,ti OR action:ab,ti OR strateg*:ab,ti OR program:ab,ti OR activit*:ab,ti) 5393 | 4274 |
| Web of science | (((((ALL=(salt)) OR ALL=(sodium)) AND ALL=((ALL=(hypertension)) OR ALL=(blood pressure))) AND ALL=(((ALL=(intake)) OR ALL=(consum*)) OR ALL=(diet))) AND ALL=((((((((ALL=(reduce*)) OR ALL=(restrict*)) OR ALL=(minimi*)) OR ALL=(eliminat*)) OR ALL=(limit)) OR ALL=(low)) OR ALL=(free)) OR ALL=(curb))) AND ALL=((((((((ALL=(polic*)) OR ALL=(intervention)) OR ALL=(initiative)) OR ALL=(regulation)) OR ALL=(action)) OR ALL=(strateg*)) OR ALL=(program)) OR ALL=(activit*)) | 5056 |
