# Supplementary material for: Effect of salt reduction interventions in lowering blood pressure: A comprehensive systematic review and meta-analysis of controlled clinical trials
Source: PLoS One. 2022 Dec 7;17(12):e0277929. doi: 10.1371/journal.pone.0277929 (PMC9728935; doi:10.1371/journal.pone.0277929)
Supplement: S2 Table — (DOCX) [file pone.0277929.s003.docx]

| **Table S2. Meta-regression on the association of intervention effect on SBP and DBP change with study quality, age category, hypertension status, study duration, and sample size** | | | | | | | | |
| --- | --- | --- | --- | --- | --- | --- | --- | --- |
|  | SBP | | | | DBP | | | |
|  | Coefficients | SE | 95% CI | P value | Coefficients | SE | 95% CI | P value |
| **Education** |  |  |  |  |  |  |  |  |
| ***Quality*** | 0.22 | 0.37 | -0.57, 1.02 | 0.563 | 0.05 | 0.32 | -0.62, 0.73 | 0.875 |
| ***Age (<25 year versus others)*** | -2.64 | 0.98 | -4.71, -0.56 | 0.016 | -2.35 | 0.84 | -4.14, -0.56 | 0.013 |
| ***Hypertension status*** ***(normotensive versus others)*** | 0.35 | 1.19 | -3.67, 4.37 | 0.857 | 1.42 | 1.61 | -1.96, 4.81 | 0.388 |
| ***Duration*** | -0.18 | 0.11 | -0.42, 0.06 | 0.135 | -0.02 | 0.11 | -0.25, 0.22 | 0.886 |
| ***Sample size*** | -0.001 | 0.002 | -0.007, 0.004 | 0.714 | -0.000 | 0.003 | -0.006, 0.004 | 0.700 |
| **Salt substitute** |  |  |  |  |  |  |  |  |
| ***Quality*** | -0.63 | 1.48 | -3.87, 2.60 | 0.678 | -1.04 | 1.22 | -3.63, 1.56 | 0.402 |
| ***Age (<25 year versus others)*** | 0.67 | 1.21 | -1.98, 3.33 | 0.587 | 1.76 | 0.83 | -0.07, 3.59 | 0.058 |
| ***Hypertension status (normotensive versus others)*** | 1.27 | 1.25 | -1.45, 4.01 | 0.329 | 0.86 | 1.09 | -1.53, 3.25 | 0.448 |
| ***Duration*** | 0.07 | 0.03 | -0.01, 0.14 | 0.055 | 0.04 | 0.03 | -0.03, 0.11 | 0.293 |
| ***Sample size*** | 0.002 | 0.001 | -0.001, 0.003 | 0.062 | 0.001 | 0.001 | -0.002, 0.003 | 0.077 |
| **Self-help materials** |  |  |  |  |  |  |  |  |
| ***Quality*** | -0.05 | 0.69 | -1.69, 1.58 | 0.936 | -0.19 | 0.47 | -1.35, 0.96 | 0.701 |
| ***Age (<25 year versus others)*** | -1.26 | 1.43 | -4.56, 2.03 | 0.403 | -1.47 | 1.01 | -3.87, 0.93 | 0.191 |
| ***Hypertension status*** ***(normotensive versus others)*** | -0.39 | 1.24 | -3.27, 2.47 | 0.757 | -0.48 | 0.87 | -2.55, 1.60 | 0.603 |
| ***Duration*** | -2.52 | 2.16 | -7.51, 2.45 | 0.276 | -1.33 | 1.43 | -4.73, 2.06 | 0.384 |
| ***Sample size*** | -0.007 | 0.026 | -0.06, 0.05 | 0.979 | 0.03 | 0.01 | -0.01, 0.06 | 0.090 |
| CI: Confidence interval, SBP: Systolic blood pressure, DBP: Diastolic blood pressure | | | | | | | | |
